# Impacts of host phylogeny, diet, and geography on the gut microbiome of rodents

**DOI:** 10.1371/journal.pone.0316101

**Published:** 2025-01-16

**Authors:** Sufia Akter Neha, John D. Hanson, Jeremy E. Wilkinson, Robert D. Bradley, Caleb D. Phillips

**Affiliations:** 1 Department of Biological Sciences, Texas Tech University, Lubbock, Texas, United States of America; 2 Blackhawk Genomics, Lubbock, Texas, United States of America; 3 PacBio, Menlo Park, California, United States of America; 4 Natural Science Research Laboratory, Museum of Texas Tech University, Lubbock, Texas, United States of America; Central University of Kerala, INDIA

## Abstract

Mammalian gut microbial communities are thought to play a variety of important roles in health and fitness, including digestion, metabolism, nutrition, immune response, behavior, and pathogen protection. Gut microbiota diversity among hosts is strongly shaped by diet as well as phylogenetic relationships among hosts. Although various host factors may influence microbial community structure, the relative contribution may vary depending on several variables, such as taxonomic scales of the species studied, dietary patterns, geographic location, and gut physiology. The present study focused on 12 species of rodents representing 3 rodent families and 3 dietary guilds (herbivores, granivores, and omnivores) to evaluate the influence of host phylogeny, dietary guild and geography on microbial diversity and community composition. Colon samples were examined from rodents that were collected from 7 different localities in Texas and Oklahoma which were characterized using 16S rRNA gene amplicon sequencing targeting the V1-V3 variable regions. The microbiota of colon samples was largely dominated by the family Porphyromonadaceae (*Parabacteriodes*, *Coprobacter*) and herbivorous hosts harbored richer gut microbial communities than granivores and omnivores. Differential abundance analysis showed significant trends in the abundance of several bacterial families when comparing herbivores and granivores to omnivores, however, there were no significant differences observed between herbivores and granivores. The gut microbiotas displayed patterns consistent with phylosymbiosis as host phylogeny explained more variation in gut microbiotas (34%) than host dietary guilds (10%), and geography (3%). Overall, results indicate that among this rodent assemblage, evolutionary relatedness is the major determinant of microbiome compositional variation, but diet and to a lesser extent geographic provenance are also influential.

## Introduction

Gut microbiomes, which are communities of microbes residing within hosts’ digestive systems, are important to several aspects of biology including digestion, metabolism, nutrition, immune response, behavior, and pathogen invasion [1–5]. Considering the importance of gut microbiota for host health and fitness, researchers have examined the factors that influence its diversity and composition. An emerging view from comparisons across species is that gut microbiome structure is shaped in large part by host phylogeny; such patterns have been observed in a wide variety of taxa such as mammals, birds, and invertebrates [6–9]. The effect of host phylogeny results in closely related hosts tending to have higher similarity in gut microbiota composition than distantly related hosts [10,11], a phenomenon referred to as phylosymbiosis [10,12,13]. Giant pandas are an extreme example of the apparent strength of host phylogeny; although they have altered their diet drastically in the past to herbivory, their gut microbiome more closely resembles that of their carnivore relatives [14].

Diet is another strong predictor of gut microbiome diversity and composition. For example, Ley et al. 2008 [15] found that not only host phylogeny but also diet strongly explained microbial flora in 13 taxonomic orders of mammals. Furthermore, dietary preference has been predominantly linked to host gut morphology with complex gastrointestinal tracts in herbivorous mammals required to digest plant-derived materials compared to the simple gut systems in carnivores, thus allowing herbivores to harbor diverse bacterial communities [15]. Although both host phylogeny and diet may influence microbial community composition, they are not independent and their relative contribution may vary depending on a number of variables, such as taxonomic scale of the species studied, host habitat, dietary variability and gut morphology [9,16–18]. Recent studies showed that the microbiota of closely related species, such as mice, voles, and shrews, inhabiting similar habitats and eating similar foods, are more similar than that of the same species inhabiting different habitats and eating different foods [19]. In contrast, distantly related taxa with similar diets tend to have similar microbial communities [20]. For example, folivorous primates with overlapping diets in different geographic locations display similarities in their gut microbiota [21].

Gut microbiome composition in mammals may further be influenced by geography [17,22–24]. Geographic location has been shown to affect the composition of gut microbial communities of humans [25], rodents [22,26], bats [9], and insects [27]. For example, sampling locality accounted for 16% variation in the gut microbiota of black-tailed prairie dogs in Texas [22]. Dispersal abilities of microbes and direct contact between hosts may have a significant influence on microbiome variation [17,24]. In a homogenous environment with limited dispersal, we would anticipate hosts living nearby to have a more similar gut microbial structure compared to hosts living further away [3]. Geographic locations vary in environmental features such as topography, vegetation, and altitude which may also shape the gut microbiome composition. For an example of continuous geographic variation, it was found that the gut microbiota of wild mice changed significantly over an elevational gradient varying in temperature, air pressure, and oxygen concentration [26].

A more comprehensive understanding of gut microbial community structure in wild mammals requires the interaction of various factors that are critical to inform the symbiotic relationship between hosts and their microbes. There have been multiple studies investigating the mechanisms influencing gut microbial communities, but most of the existing research concentrated on a single host species at multiple geographic locations or several species at one location [7,22,28,29]. A few studies have investigated the combined effects of host phylogeny, diet, and geographical provenance on microbial communities in wild mammals [6,7,9], particularly rodent populations that are widely distributed [30,31].

Herein, we examined the microbiomes of 12 rodent species occurring in Texas and Oklahoma including Pinon deermouse (*Peromyscus truei*), brush deermouse (*Peromyscus boylii*), cotton deermouse (*Peromyscus gossypinus*), white-footed deermouse (*Peromyscus leucopus*), northern rock deermouse (*Peromyscus nasutus*) Mexican woodrat (*Neotoma mexicana*), white-toothed woodrat (*Neotoma leucodon*), eastern woodrat (*Neotoma floridana*), hispid pocket mouse (*Chaetodipus hispidus*), hispid cotton rat (*Sigmodon hispidus*), Attwater’s pocket gopher (*Geomys attwateri*), Baird’s pocket gopher (*Geomys breviceps*). These rodent species represent three families (Cricetidae, Geomyidae, and Heteromyidae) and three 3 dietary guilds (herbivores, granivores, and omnivores). The objectives of this study were to characterize the gut microbiota profiles among these rodents and examine the relative effects of host phylogeny, dietary guild and geographic location on gut microbial community composition. Overall, this study will enhance our understanding of the factors shaping gut microbial communities across diverse host species, which is crucial for improving conservation efforts and informing management practices for these species.

## Methods

### Rodents sampling

**Fig 1:**
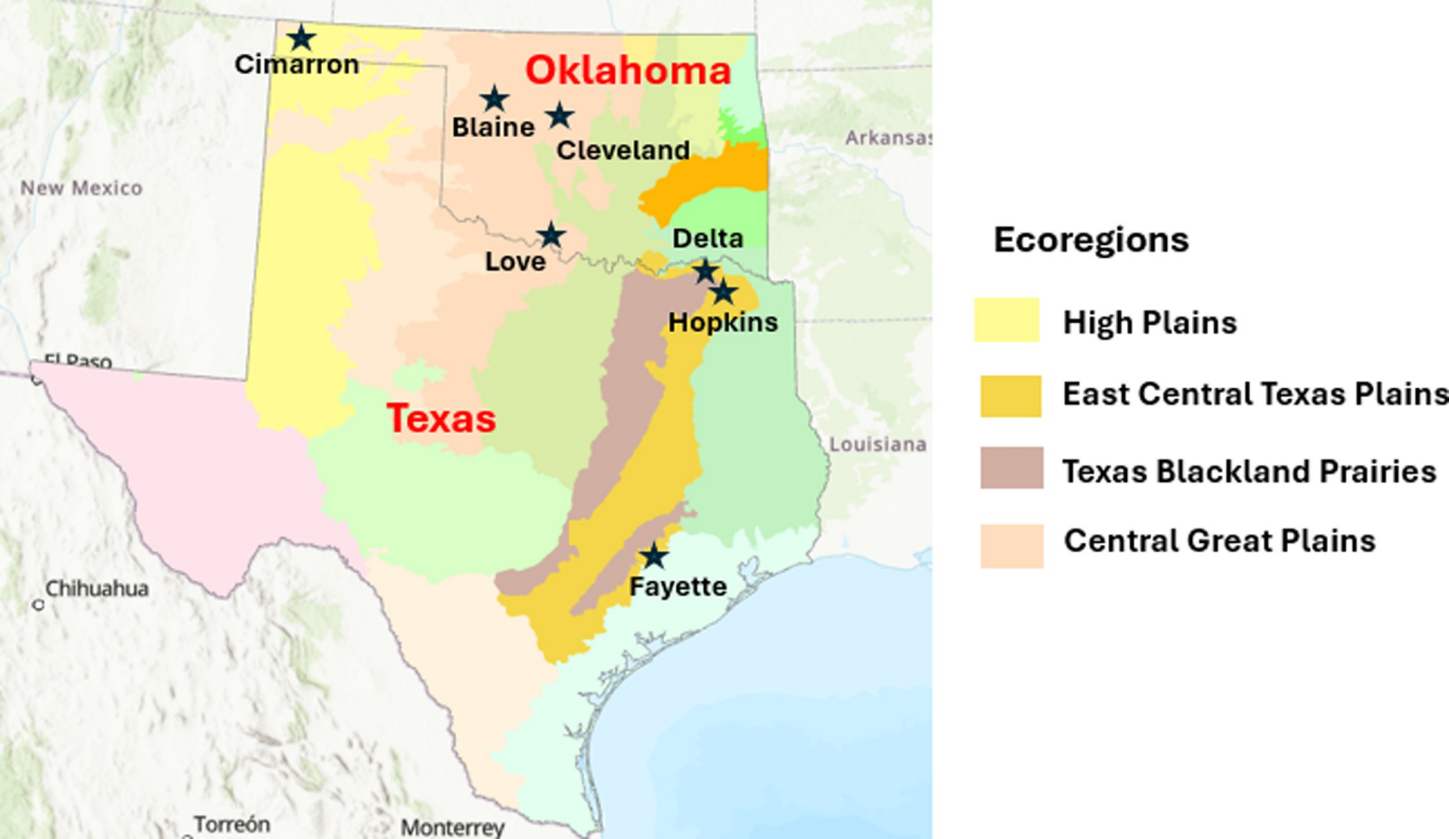
A map of ecoregions of Texas and Oklahoma (geospatial data were downloaded from US environmental protection agency [33]) showing the sampling locations of the twelve species of rodents. The map was created with ArcGIS Pro (v 2.9.0).

A total of 71 gut samples were collected from 12 species of rodents over a two-week field period in Texas and Oklahoma in 2016. Among them, 22 samples were obtained from Fayette County, 14 samples from Hopkins County, 5 samples from Delta County, 25 samples from Cimarron County, 5 samples from Cleveland County, 2 samples from Love County, and 1 sample from Blaine County (Fig 1 and Table 1). The geographic regions where the samples were collected vary in climate, rainfall, and vegetation. Texas counties like Fayette, Hopkins, and Delta have a humid subtropical climate with around 35–45 inches of annual rainfall, supporting a mix of grasslands, oak-hickory forests, and agricultural areas. In contrast, Oklahoma counties like Cimarron experience a semi-arid climate with less rainfall (around 17 inches) and are dominated by shortgrass prairies. The other Oklahoma counties (Cleveland, Love, and Blaine) have more rainfall and support a combination of grasslands, woodlands, and farmland. Sherman live traps baited with oats were placed in the trapline about 2–10 m apart each night. The following morning individuals were euthanized with isoflurane for 1–2 minutes. The animals were dissected, and colon samples were taken from each captured individual, which was stored in liquid nitrogen and then kept at -80°C for later analysis. Specimens were collected using the methods outlined in the American Society of Mammalogists guidelines [32]. The sampling protocol received approval from the Texas Tech University Institutional Animal Care and Use Committee (IACUC permit #: 14006–01) and Scientific Permit of Texas (#SPR-0393-593).

**Table 1:** Sample information of rodent species. Numbers inside parentheses are sample sizes obtained from each locality.

Host species	Dietary guild	Number of samples	State	Locality (County)
*C*. *hispidus*	Granivore	4	Texas	Fayette
*G*. *attwateri*	Herbivore	4	Texas	Fayette
*G*. *breviceps*	Herbivore	4	Oklahoma	Love (2), Cleveland (2)
*N*. *floridana*	Herbivore	7	Texas, Oklahoma	Fayette (3), Delta (3), Cleveland (1)
*N*. *leucodon*	Herbivore	5	Oklahoma	Cimarron
*N*. *mexicana*	Herbivore	5	Oklahoma	Cimarron
*P*. *boylii*	Omnivore	4	Oklahoma	Cimarron
*P*. *gossypinus*	Omnivore	6	Texas	Hopkins (4), Delta (2)
*P*. *leucopus*	Omnivore	11	Texas, Oklahoma	Fayette (4), Hopkins (1), Cleveland (2), Blaine (1), Cimarron (3)
*P*. *nasutus*	Omnivore	5	Oklahoma	Cimarron
*P*. *truei*	Herbivore	3	Oklahoma	Cimarron
*S*. *hispidus*	Herbivore	16	Texas	Fayette (7), Hopkins (9)

### DNA extraction and library preparation

DNA was extracted from the distal portion of the colon containing feces using Qiagen DNeasy Blood and Tissue Kit (Qiagen, Valencia, CA, USA) according to the manufacturer’s guidelines. Samples were characterized by targeting V1-V3 variable regions of 16S rRNA gene as described by [34]. Briefly, the forward primer was designed with the following sequence (5′-3′): the Illumina i5 adapter (AATGATACGGCGACCACCGAGATCTACAC), an 8–10 bp barcode, a primer pad, and 28F (GAGTTTGATCNTGGCTCAG) and the reverse primer was designed with the following sequence (5′-3′): the Illumina i7 adapter (CAAGCAGAAGACGGCATACGAGAT), an 8–10 bp barcode, a primer pad, and 519R (GAGTTTGATCNTGGCTCAG). Amplifications were carried out using 1 μl of each 5μM forward and reverse primer along with 1.0 μl of template DNA. The thermal conditions were as follows: 95°C for 5 minutes, 35 cycles of 94°C for 30 seconds for initial denaturation, followed by annealing at 54°C for 40 seconds; extension at 72°C for 1 minute, and a final extension at 72°C for 10 minutes and a final hold at 4°C. The amplification products were visualized using eGels (Life Technologies, Grand Island, New York) to determine library quantity and AMPure XP beads were used for reaction cleanup. After pooling equimolar libraries, the libraries were quantified with the Qubit 2.0 Fluorometer (Thermo Scientific, USA). Libraries were sequenced for 2 × 300 bp reads with Illumina MiSeq protocol (Illumina Inc, San Diego, CA, USA) at RTLGenomics (Lubbock, TX, USA). Raw sequence data has been deposited in the NCBI Sequence Read Archive (SRA) under the BioProject ID PRJNA1107948.

### Bioinformatics

Read pairs were merged using PEAR [35], and sequences with more than one estimated error were eliminated using USEARCH fastq-filter function. The remaining sequences were clustered into zero-radius operational taxonomic units (ZOTUs) using the denoising algorithm UNOISE3 [36], and USEARCH was used to build the OTU table from the ZOTUs. ZOTUs detect and correct sequencing errors, generating sequence variants that are 100% identical. They offer higher resolution than OTUs by distinguishing sequences that differ by even a single nucleotide, enabling a more precise and accurate representation of microbial diversity. Representative ZOTU sequences were compared to the SILVA database (v. 123) [37] to assign taxonomy. SSU-ALIGN [38] was used to create the ZOTU alignment and a phylogenetic tree to summarize the evolutionary relationship among ZOTUs was estimated using FastTree2 [39].

### Statistical analyses

The ZOTU, taxonomy tables and phylogeny were combined into a relational phyloseq object [40]. Analyses were conducted with the following packages in R [41]: phytools [42], ape [43], vegan [44], picante [45], scales [46], reshape2 [47], pairwiseAdonis [48], lme4 [49], and ggplot2 [50].

Sequencing effort was summarized using scaled ranked subsampling (SRS) to normalize the ZOTU counts to the size of the smallest sample in the dataset, employing SRS R package [51]. The rarefied dataset was further used for diversity analyses. Good’s coverage was also used to make sure that samples were well represented, and what proportion of total species present in the community were detected in the sample [52]. Alpha diversity was summarized as Hill_0_, which is number of observed species (i.e., richness); Hill_1_, which is the exponentiation of Shannon diversity, as well as Faith’s phylogenetic diversity [53] which summarizes alpha diversity proportional to branch length of the community phylogeny observed in each sample. The Shapiro-Wilk test was used to test for normality, and all diversity estimates appeared normally distributed (p > 0.05). Also, homogeneity of variance testing yielded non-significance of variance differences (host species: F = 1.555, p = 0.137; dietary guild: F = 1.834, p = 0.158; geographic location: F = 1.252, p = 0.616) further supporting the use of parametric statistics. Multiple regression was used to simultaneously evaluate the effects of host species, dietary guild, and geographic location on alpha diversity measures. This was followed by post-hoc pairwise testing (Tukey’s HSD) to determine which pairwise comparisons were significant. Phylogenetic non-independence was controlled by implementing the phy.anova function in the geiger package [54].

Variation in community composition was summarized using unweighted and weighted UniFrac phylogenetically-informed distances. Unweighted UniFrac distance [55] is based on presence/absence of species, whereas weighted UniFrac accounts for relative abundance of taxa. Unweighted UniFrac may be optimal in cases when compared communities broadly differ in occurrence of species, and weighted UniFrac will be more discriminating in cases where compared communities share many species but differ in proportions. To determine predictors of community composition, distance-based multivariate analysis of variance (function dbrda) was used, and predictors in this model included individual-level pairwise genetic distances of host species in addition to host species, dietary guild and locality. Post-hoc pairwise testing was used to determine which pairwise comparisons were different. To visualize the grouping across samples, redundancy analysis (RDA) was used. Because significant multivariate analysis of variance results can arrive from group differences in location or spread in multivariate space, Euclidean distances from group centroids resulting from the RDA were also computed and differences in dispersion were assessed with ANOVA. The relative abundance of bacterial taxa was visualized using bar plots.

Linear Discriminant Analysis Effect Size (LEfSe) was employed to identify differentially abundant families between dietary guilds. This analysis utilized the microbiomeMarker package [56] and included the Kruskal-Wallis sum-rank test to determine significant differential abundance, set at a significance level of p = 0.05. Subsequently, the analysis employed Linear Discriminant Analysis (LDA) to estimate effect sizes, represented as log(10) values [57]. The results were visualized to highlight taxa demonstrating an LDA of at least 2 for effect size.

Phylogenetic relationships among hosts were determined based on the mitochondrial cytochrome-b gene (1143 bp). In total, 127 sequences were acquired from NCBI GenBank (S1 Table). *Sylvilagus holzneri* was used as the outgroup for the phylogeny. The resulting sequences were aligned using MUSCLE [58] for downstream analyses. The RAxML [59] was employed to eliminate similar sequences from the dataset, resulting in a final set of 111 sequences for phylogenetic analyses. jModelTest-2.1.10 [60] evaluated 88 maximum likelihood models and the corrected Akaike information criterion [61] was used to determine the best-fit model for the cytochrome-b dataset. HKY+I [62] was chosen as the best-fit model (-lnL = 12291.279095) and the following parameters for likelihood analysis were used: base frequencies (A = 0.30253, C = 0.284131, G = 0.128856, and T = 0.284479). Bootstrap support (BS) values with 1,000 iterations were used [63], and BS ≥ 65 determined moderate to strong nodal support. Bayesian inference was performed (BI) in MrBayes [64] to generate the posterior probability values (PPV). In Bayesian analysis, we used GTR + I + Γ model and estimated parameters based on the following: 4 Markov chains with two independent runs, 10 million generations, and 1,000 generation sample frequency. Based on likelihood scores, 10 percent of the first 1,000,000 trees were removed, and 50% majority rule consensus tree was constructed. The PPV≥ 0.95 indicated strong nodal support [65]. Host dietary information was obtained from published articles (S2 Table).

We illustrated the phylosymbiosis findings by creating host species phylogeny and contrasted it with a dendrogram of gut microbiota similarity using hierarchical clustering resulting from Unweighted UniFrac distances. The correlation between host evolutionary lineage and the microbial community was evaluated using partial mantel test.

## Results

### Rodents gut microbiota profile

There were 1,461,683 raw sequences from 71 gut samples, averaging 20,587 reads/sample (range = 9,034–60,726; SD = 9,378). Upon quality filtering and denoising, 1,257,245 non-chimeric sequences yielded an average of 17,707 reads/sample (range = 7,689–52,041; SD = 8,005). In total, 15,736 ZOTUs were detected across all samples. Rarefaction curves of Hill_0_ and Hill_1_ indicated sequencing effort was sufficient to adequately characterize bacterial communities and reached a plateau at the sequence depth of 6,000 (S1 Fig). Good’s coverage values averaged 95.91 ± 6.44 across all samples which also supported that sample coverage was sufficient to compare microbial communities.

### Microbiome composition

**Fig 2:**
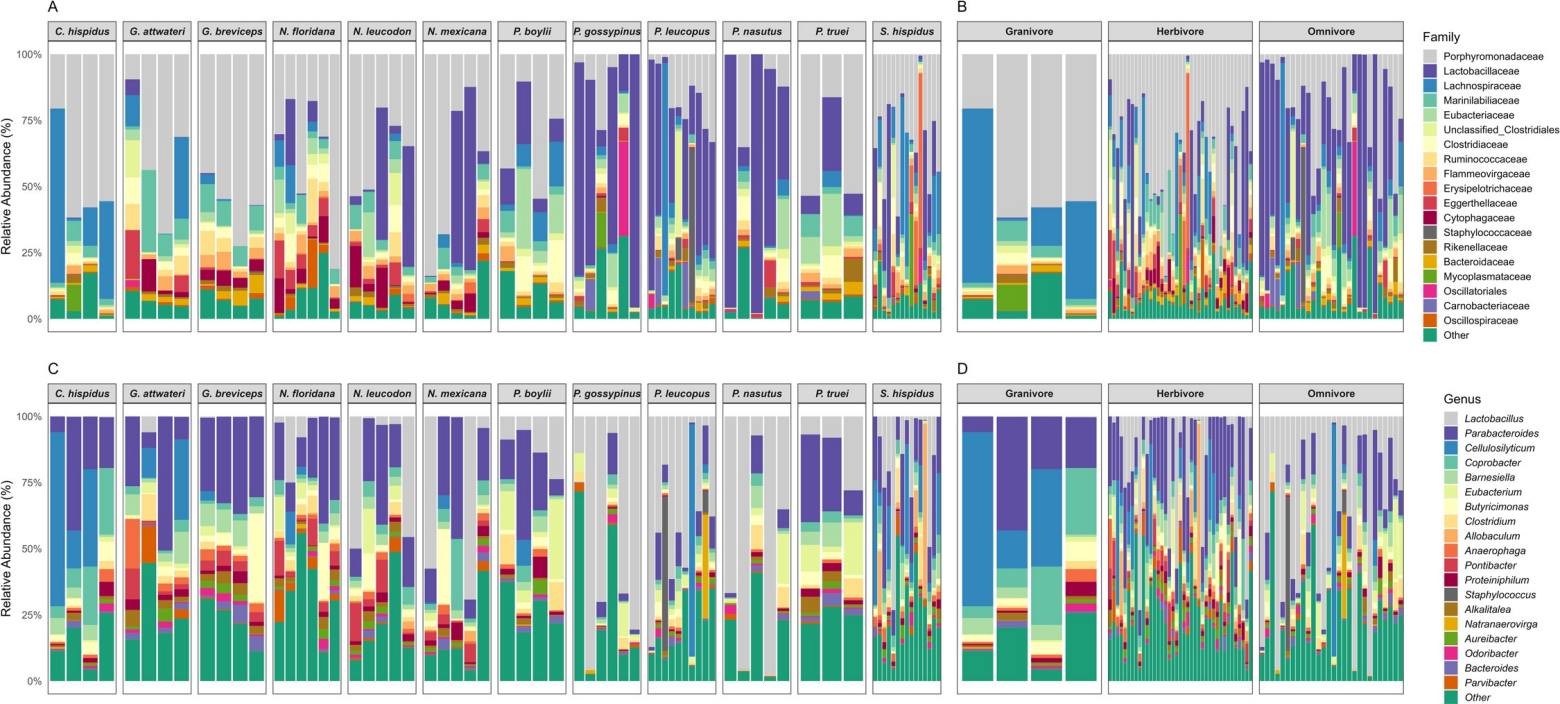
Stacked bar plots showing the relative abundance of top 20 bacterial families and genera grouped by host species (A and C) and dietary guild (B and D).

A total of 120 families, 281 genera, and 553 species of bacteria were observed study wide. Approximately 80% of the gut microbiota was represented by the top ten families including Porphyromonadaceae (40.0%), Marinilabiliaceae (6.7%), Lachnospiraceae (6.1%), Lactobacillaceae (5.0%), Clostridiaceae (4.7%), Eubacteriaceae (4.3%), Ruminococcaceae (3.9%), Eggerthellaceae (3.4%), Flammeovirgaceae (3.1%) and Unclassified_Clostridiales (3.0%). The most abundant genera were *Parabacteroides* (22.1%), *Lactobacillus* (5.0%), *Coprobacter* (4.8%), *Barnesiella* (4.3%), and *Eubacterium* (4.2%). Distributions of bacteria across host species, and dietary guilds are provided in Fig 2.

### Host phylogeny and testing for phylosymbiosis

**Fig 3:**
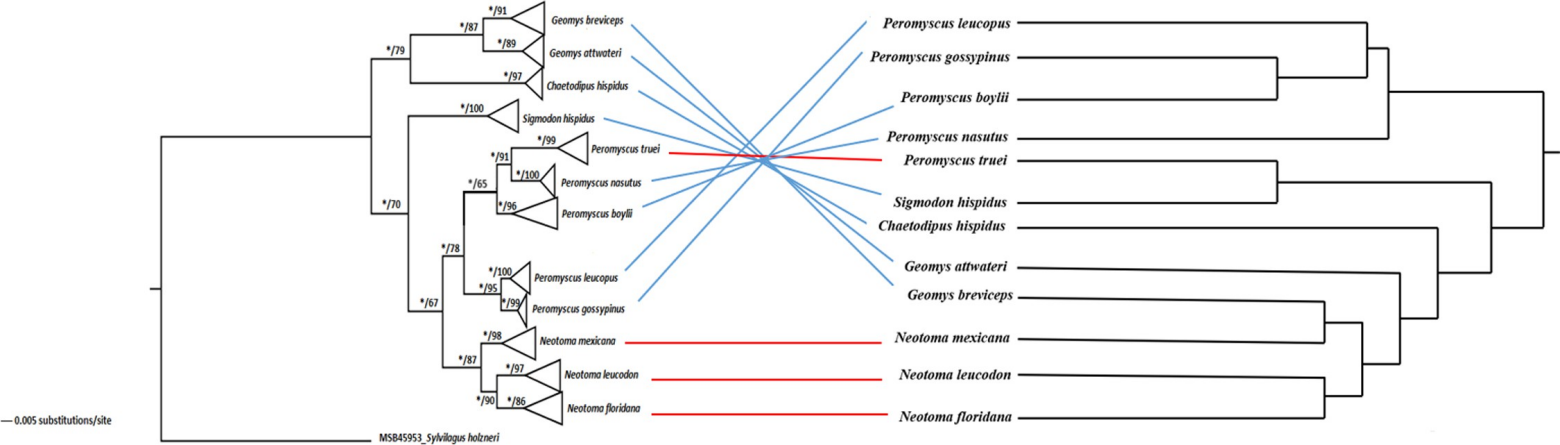
Relationship between host phylogeny and gut microbiota similarity. (A) Bayesian phylogeny of 12 host species based on mitochondrial cytochrome-b. Node posterior probability values (PPV ≥ 0.95) are indicated by asterisks, and likelihood bootstrap support values occur after the slashes (BS ≥ 0.65). (B) Dendrogram showing similarities in gut microbiota using hierarchical clustering based on unweighted UniFrac distances. Lines connect species positions in both trees.

A total of 127 complete cytochrome-b sequences (1,143 bp) were obtained from GenBank, representing the 12 host species. The phylogenetic analysis of host sepcies produced similar tree topologies when using Maximum Likelihood (ML) and Bayesian Inference (BI), but only the BI topology is presented here (Fig 3). Based on BI analysis, two major clades were identified as supported. *G*. *attwateri* and *G*. *breviceps* constituted a strongly supported clade (PPV > 95%, nucleotide distance of 0.13; see S3 Table), and this clade was identified as the sister group to *C*. *hispidus*. *S*. *hispidus* formed a sister clade to another large and well-supported monophyletic clade containing *P*. *leucopus*, *P*. *gossypinus*, *P*. *nasutus*, *P*. *boylii*, *P*. *truei*, *N*. *leucodon*, *N*. *floridana*, and *N*. *mexicana*. However, the nodal support for *P*. *nasutus*, *P*. *boylii*, *P*. *truei* was moderately supported in ML analysis (BS = 65) but strongly supported in BI analysis (PPV > 95%) (Fig 3). The dendrogram depicting gut microbiota dissimilarity was obtained from hierarchical clustering based on unweighted UniFrac distances by averaging the OTU counts for each host species (Fig 3). The explanatory power of host phylogeny was assessed with a partial mantel test through which a significant effect of host phylogeny on microbiome was observed (r = 0.32, p < 0.001).

### Microbial alpha diversity

**Fig 4:**
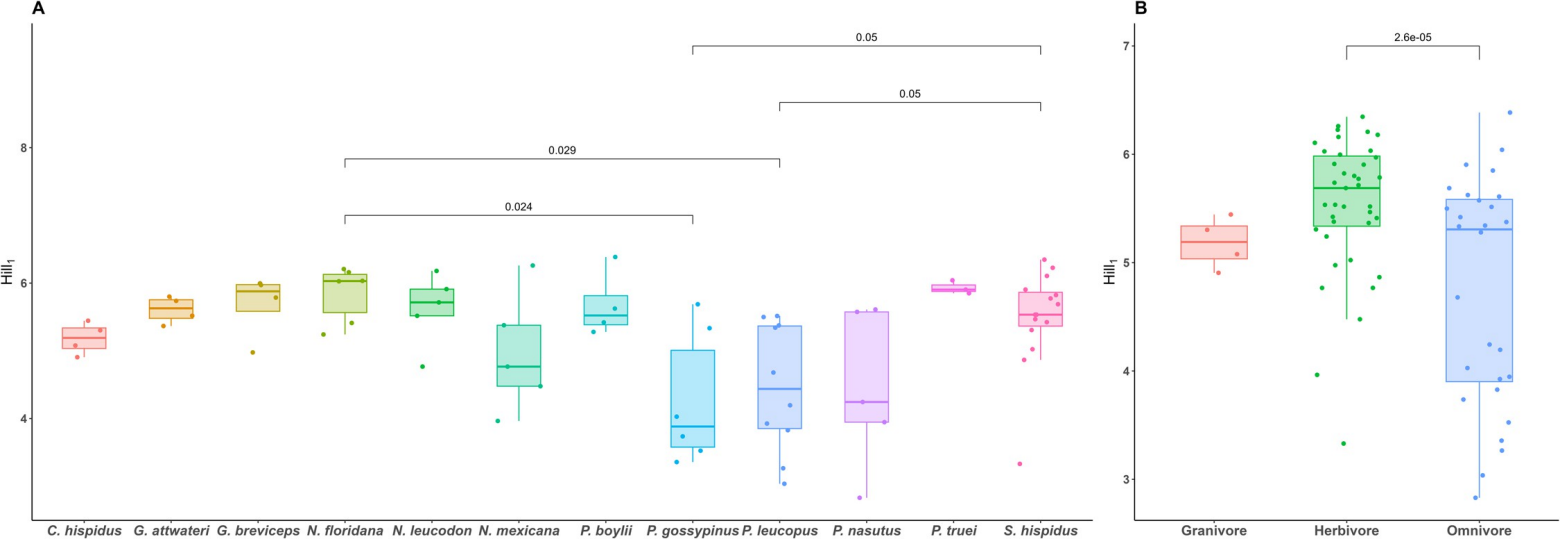
Boxplots illustrating Hill_1_ distributions grouped by (A) host species; and (B) dietary guilds. The boxes of the boxplots are defined by 1^st^ and 3^rd^ quartiles, the horizontal lines within boxes are medians, and whiskers calculated as 1.5 times the interquartile range. Benjamini-Hochberg adjusted p-values given by Tukey’s HSD tests to control for multiple comparisons.

The alpha diversity of microbial communities was 739.85±31.29 for Hill_0,_ 5.23±0.11 for Hill_1,_ and 15.75±0.49 for Faith’s phylogenetic diversity (S4 Table). Hill_0_ was not significantly explained by host species, dietary guild, or locality in the multiple regression model while controlling for phylogenetic non-independence (host species: F = 1.554, P = 0.137; dietary guild: F = 2.166, P = 0.123; locality: F = 1.274, P = 0.273). However, Hill_1_ was found to be significantly explained by host species and dietary guild but not by locality (host species: F = 3.681, P < 0.001; dietary guild: F = 11.53, P < 0.001; locality: F = 1.826, P = 0.089; Fig 4A–4C). Post-hoc pairwise testing of Hill_1_ revealed that *N*. *floridana* and *S*. *hispidus* had significantly higher Hill_1_ than *P*. *leucopus* and *P*. *gossypinus* (BH adjusted p-values <0.05; Tables 2 and S5), respectively, and herbivores had significantly higher Hill_1_ compared to omnivores (BH adjusted p-values <0.05, S5 Table). Faith’s phylogenetic diversity was not explained by host species, dietary guild, or locality (host species: F = 1.322, P = 0.235; dietary guild: F = 0.446, P = 0.652; locality: F = 1.285, P = 0.283).

**Table 2:** Microbial alpha diversity summary statistics grouped by host species and dietary guild. Mean values are provided with their corresponding standard deviations (±) for each metric.

Host species	Samples	Total reads	Reads after filtering	Hill_0_	Hill_1_	Faith’s PD
*C*. *hispidus*	n = 4	16,564±2633	14,404±2265	582.7±48.8	5.21±0.12	14.08±0.39
*G*. *attwateri*	n = 4	19,411±5060	16,526±4199	648±42.1	5.65±0.08	13.72±1.25
*G*. *breviceps*	n = 4	23,519±3581	20,028±3068	739±48.6	5.71±0.23	15.11±1.05
*N*. *floridana*	n = 6	15,309±1197	13,210±1005	756±78.3	5.89±0.16	16.35±1.58
*N*. *leucodon*	n = 5	26,034±9374	22,308±8029	818±66.5	5.64±0.24	16.91±1.24
*N*. *mexicana*	n = 5	16,021±2081	13,783±1800	697±96.7	5.01±0.41	14.09±1.41
*P*. *boylii*	n = 4	22,166±3400	18,839±3002	931±127.8	5.70±0.26	19.93±1.85
*P*. *gossypinus*	n = 6	17,133±1781	14,782±1574	537±110.5	4.31±0.41	14.41±2.15
*P*. *leucopus*	n = 10	28,930±3490	25,005±2955	701±102.7	4.51±0.31	16.76±1.54
*P*. *nasutus*	n = 5	19,729±3285	17,106±2777	602±205	4.45±0.54	14.08±3.57
*P*.*truei*	n = 3	21,061±6315	17,825±5268	1039±59.7	5.97±0.18	20.92±0.81
*S*. *hispidus*	n = 15	18,598±1426	16,034±1233	828±59.7	5.52±0.19	15.10±0.80
**Dietary guild**						
Granivore	n = 4	16,564±2633	14,404±2265	582.7±48.8	5.21±0.12	14.08±0.39
Herbivore	n = 42	19,429±1428	16,666±1216	790±32.12	5.59±0.09	15.66±0.51
Omnivore	n = 25	23,176±1907	19,985±1632	679.9±67.86	4.64±0.21	16.17±1.11

### Microbial beta diversity

**Fig 5:**
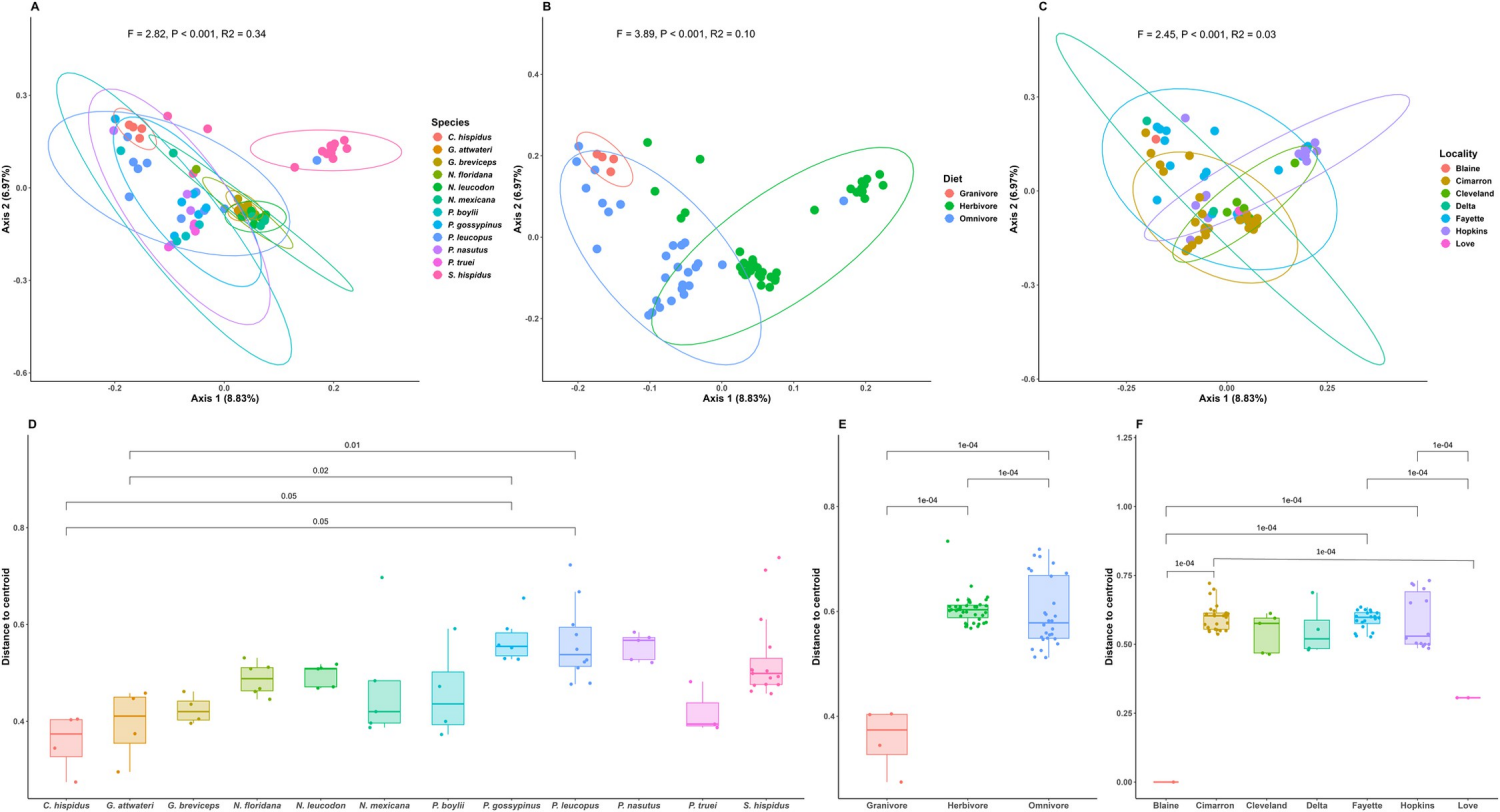
Beta diversity compositional variation summarized using dbrda with unweighted UniFrac distance among (A) host species; (B) dietary guilds and (C) locality. Corresponding PERMANOVA results are provided. Each data point corresponds to an individual sample, and ellipses indicate group 95% confidence distributions. Group dispersion based on unweighted UniFrac distance among (D) host species; (E) dietary guild and (F) locality. The boxes of the boxplots are defined by 1^st^ and 3^rd^ quartiles, the horizontal lines within boxes are medians, and whiskers calculated as 1.5 times the interquartile range. Test values are Benjamini-Hochberg adjusted p-values given by Tukey’s HSD tests to control for multiple comparisons.

To investigate determinants of bacterial community composition, we used dbRDA incorporating host phylogenetic distance among host species in combination with dietary guild and locality as predictors. Based on dbRDA, variance in unweighted UniFrac was significantly explained by host phylogeny (F = 2.82, R^2^ = 0.34, P < 0.001), host species (F = 4.24, R^2^ = 0.20, P < 0.001), dietary guild (F = 3.27, R^2^ = 0.10, P < 0.001), and geography (F = 2.452, R^2^ = 0.03, P < 0.001). Post-hoc pairwise testing among species, dietary guild, and locality revealed that most pairwise comparisons were significant (p < 0.001, S6 Table). The ordination illustrates how axes 1 and 2 tend to separate samples consistent with significant predictor variables (Fig 5A–5C). Significant differences in dispersion of samples from the group centroids in multivariate space was also observed for host species (F = 4.22, R^2^ = 0.27, p < 0.001, Fig 5D), dietary guild (F = 54.82, R^2^ = 0.22, p < 0.001, Fig 5E) and locality (F = 20.62, R^2^ = 0.08, p < 0.001, Fig 5F). Pairwise comparisons of host species showed significant dispersion for *C*. *hispidus* and *G*. *attwateri* relative to *P*. *gossypinus* and *P*. *leucopus* (Fig 5D). All pairwise comparisons between dietary guilds revealed significant differences (Fig 5E). For locality, Blain and Love counties exhibited significantly different dispersion compared to Hopkins, Fayette, and Cimarron counties (Fig 5F). However, no comparisons of compositional variance in weighted UniFrac were significant for any predictor variable (P > 0.05).

### Differential abundance

**Fig 6:**
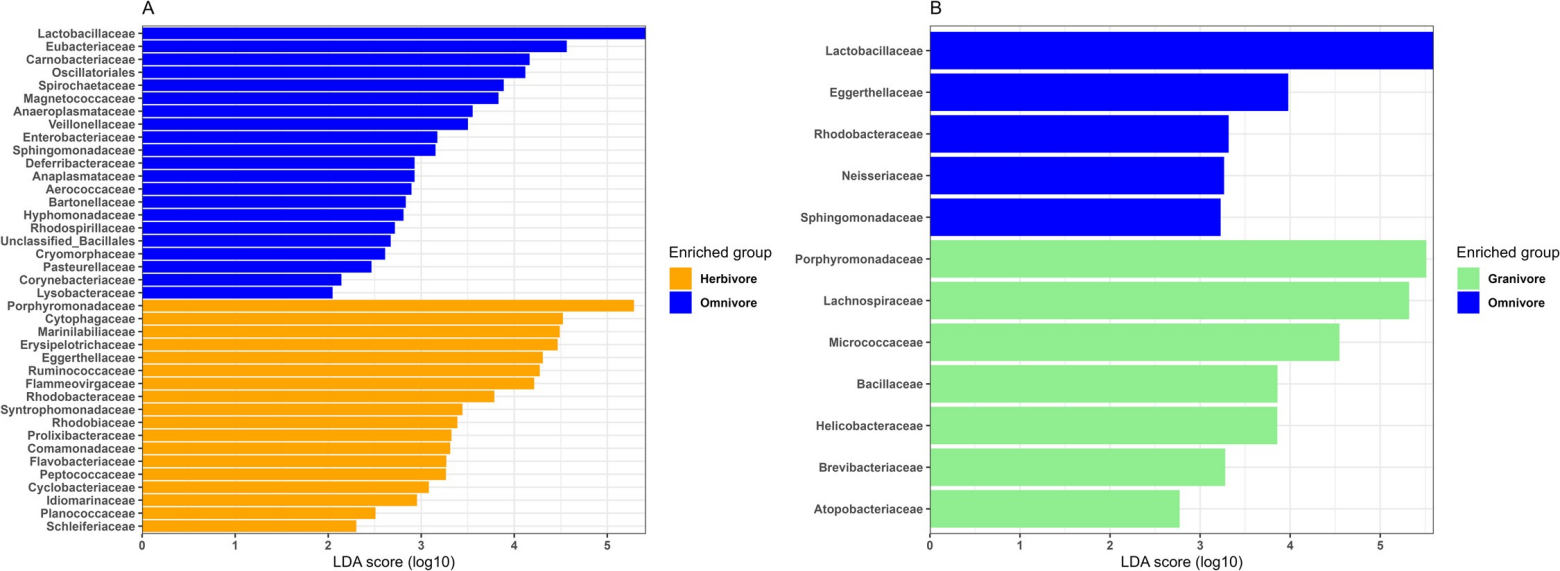
Log10 abundance differences for significant bacterial families between (A) herbivores and omnivores, and (B) granivores and omnivores.

The relative abundance of bacterial taxa was examined at the family level to identify any differentially abundant taxa between dietary groups. LEfSe analysis revealed that Lactobacillaceae was notably more abundant in omnivorous groups (71.6%) (Fig 6A), whereas Porphyromonadaceae predominated in granivores and herbivores (Fig 6A and 6B), representing 59.4% and 48.7%, respectively. However, no significant differences in bacterial abundance were observed between herbivore and granivore groups. Further LEfSe analysis with an LDA score > 2 highlighted Marinilabiliaceae, Erysipelotrichaceae, and Ruminococcaceae as the taxa contributing most to dissimilarity in herbivores, whereas Eubacteriaceae, Carnobacteriaceae, and Oscillatoriales were identified as the main contributors to dissimilarity in omnivores. Granivores exhibited a higher proportion of Lachnospiraceae, Micrococcaceae, and Helicobacteraceae.

## Discussion

This study contributes to our understanding of how host phylogeny, host species, dietary guild, and geography affects gut microbial diversity and composition in rodents from a portion of the south-central US. The key findings of this study include: (i) several bacterial families showed significant differences in abundance when comparing herbivore and granivore to omnivore dietary guilds, while no significant differences were observed between herbivores and granivores; (ii) gut microbial alpha and beta diversity were jointly shaped by host phylogeny, host species, and dietary guild, but geographic provenance, at least in the context of the current study design, had a relatively weak influence; (iii) while controlling for influences of available explanatory variables host phylogenetic relatedness was the strongest predictor, explaining about 34% of overall variation in the unweighted UniFrac metric.

The most abundant bacterial families observed in this study were Porphyromonadaceae Marinilabiliaceae, Lachnospiraceae, and Lactobacillaceae. These families are abundant bacterial taxa found in other herbivorous mammals including ruminants [66,67], leaf-eating primates [68], and rodents [31,69], which may be viewed as microbiome convergence among distantly related groups. *Parabacteroides*, *Lactobacillus*, and *Coprobacter* observed in this study are also considered dominant gut microbiota found in fiber-rich diets and provide protection to the host from toxic plant metabolites and contribute to immune system functions and anti-inflammatory responses [70–74].

Results indicated that gut microbial alpha diversity varied with host taxonomy and dietary guild but not with host geographic location. Whereas neither Hill_0_ (OTU richness) nor phylogenetic diversity were explained by host species and diet, significant differences in Hill_1_ were observed among host species and dietary guilds indicated that these groups differ in the compositional evenness of their microbiomes. When comparing Hill_1_ among host species, significance was detected in two species *S*. *hispidus* and *N*. *floridana* both having higher diversity to two other species *P*. *gossypinus* and *P*. *leucopus*. Both *Peromyscus* species are omnivores, and *S*. *hispidus and N*. *floridana* are herbivores. Thus, the observed species effects may actually be related to their diet. Among host dietary guilds, diversity was higher in herbivorous rodents. This is likely because herbivores consume higher amounts of fiber-rich plant materials, harboring more fiber-digesting bacterial taxa including *Parabacteroides* and *Cellulosilyticum*. This result is congruent with other studies that support herbivorous mammals showing higher microbial diversity compared to mammals with other foraging strategies [15,75].

LEfSe analysis revealed that Families Porphyromonadaceae, Marinilabiliaceae and Lachnospiraceae were significantly more abundant in herbivores and granivores as compared to omnivores. Functionally, such families are composed of bacteria capable of breaking down cellulose and other plant polysaccharides into absorbable molecules including short-chain fatty acids thus providing host energy [76,77]. Granivores had moderate levels of microbial diversity, containing cellulolytic and fibrolytic bacteria, which are predicted to function in the breakdown of simple sugars and complex carbohydrates [75]. In fact, granivores depend upon the gut microbiota to degrade complex fibers in order to increase their digestion rate through a combination of rapid transit times and a high activity level of digestive enzymes [78]. Lactobacillaceae and Eubacteriaceae were significantly more abundant in omnivores, groups which can promote the breakdown of complex carbohydrates including cellulose and hemicellulose and are also prevalent in a protein-based diet.

Gut microbial compositional variation assessed from the perspective of unweighted, but not weighted UniFrac resulted in significant variable effects. The contrast in test results between unweighted and weighted metrics indicates that much of the effect of assessed factors are on selection for presence/absence of taxa across bacterial phylogeny, but microbiome relative abundance distributions substantially vary. However, sampling for each individual occurred at a single time point, so how bacterial lineages change in abundance over time and in response to seasonality was untested. Longitudinal studies on changing proportions of influential species could test this hypothesis. For unweighted UniFrac, significant variables by decreasing effect size were host phylogeny, host species, dietary guild, and geographic provenance. Host phylogeny explaining the most compositional variation supports an important role of co-evolutionary mechanisms. Phylosymbiosis was supported from multivariate analyses jointly considering other predictor variables, as well as in phylogenetic congruence testing. The evidence for such host phylogenetic effects on microbiomes is accumulating. For example, similar patterns have been observed for 11 species of herbivorous mammals in Masai Mara [7], 12 species of Madagascar lemurs [79], and 7 species of deer mice in the wild and captive environment [80]. The underlying mechanistic explanation for this phenomenon is probably a multifaceted combination of phenotypic traits and ecological factors. Closely related host species on average have more similar gut morphology, physiology, cell structure, and immune function [12,81]. Closely related species are also more likely to share similar ecological, social, and behavioral characteristics which can influence microbe dispersal and colonization [82,83].

In addition to host phylogeny, host species also explained additional variation. In fact, almost all pairwise comparisons between species were significantly different. Consistent with explanations for host phylogeny, species effects are consistent with on-going co-evolutionary processes within host species, species-specific ecological, behavioral or dietary effects, or some combination of these. Finally, whereas dietary guild explained aforementioned differentially abundant taxa as well as alpha and beta diversity effects, comparison of beta diversity from the perspective of intra-group variation revealed that sampled omnivores have a more variable gut microbiome. A Previous study [7] suggested this may be due to their increased dietary variability.

Dietary guild classifications, such as granivores, herbivores, and omnivores, encompass broader feeding strategies and ecological roles. Moreover, as the only representative of the Heteromyidae family in our dataset, *C*. *hispidus* may exhibit unique microbial dynamics that result from its specific evolutionary history; its microbiome may reflect ecological and environmental influences that lead to differences with herbivore and omnivore categories. While the aggregate data reveals significant differences, the individual species comparisons may lack the statistical power needed to detect differences due to shared microbiome traits among others (Fig 5D and 5E). The two omnivore species that differ from *C*. *hispidus* may have specific adaptations or dietary influences that significantly alter their microbiome composition, while other omnivores might share characteristics that dampen their variability. Moreover, the dietary guild is inherently tied to phylogeny, where closely related species often share similar dietary preferences. This relationship poses a challenge when attempting to disentangle the effects of diet from those of evolutionary history. As a result, it is difficult to determine whether the observed patterns in gut microbiota are driven primarily by dietary habits or by phylogenetic relatedness. Future studies could benefit from using phylogenetic comparative methods or including species with more divergent diets within similar phylogenetic clades to better isolate these variables.

The relatively weak influence of geography may be explained by a combination of uneven host species representation across localities and/or the spatial scale and environmental differences among localities. Locality could influence resident host microbiomes through local chance dispersal from the environment into the host’s microbiome, as well as selectively by differences in local food resource availability. Similar findings have been reported in wild mice, where gut microbial composition was influenced by geographic location, even when sampling sites were up to 100 kilometers apart [84], or when comparing mouse populations from different countries [85]. In our study, distinct ecoregions—including prairies and high plains—were defined based on their geographic locations, each characterized by varying topography and vegetation. Previous research has shown that the diversity of food sources and their proportions in diets can substantially affect regional gut microbiome differences [86,87]. The limited sample sizes from certain localities may disproportionately skew our findings, emphasizing the necessity for a more balanced sampling strategy to draw robust conclusions about locality effects on microbial communities. However, a comprehensive analysis of the diets of species across these geographical locations is beyond the scope of this study. Future research employing metabarcoding and metagenomics sequencing techniques will provide insights into specific dietary components and their correlations with gut microbiome diversity.

In conclusion, the findings from this study of 12 species of wild rodents support that host microbiomes are simultaneously shaped by many variables. Although studying assemblages of species in the wild is difficult due to uneven sampling designs, researchers’ inability to control variables, and researcher ignorance to important variables, the ability to frame comparisons in a phylogenetic context can reveal the importance of evolutionary relatedness of hosts. Although there are multiple unaccounted inputs that may explain the phylogenetic, species-specific, and dietary guild effects, their significance seems to clearly document that gut microbiome structure has considerable deterministic input as opposed to random assembly from the local pool of encountered microbes. Overall, the results of this study provide insight into the microbial community structure and how they co-vary with host phylogeny, dietary guild, and geography.

## Supporting information

S1 Fig Rarefaction curves of Hill0 (A) and Hill1 (B) estimates for host species with subsampling between 500 and 6,000 reads at a step size of 500.
(TIF)

S1 Table Cytochrome-b sequences of twelve species of hosts including one outgroup species obtained from GenBank. GenBank accession numbers and museum voucher numbers used in this study are listed.
(PDF)

S2 Table Dietary profiles for the twelve rodent species included in this study. (PDF)

S3 Table Pairwise genetic distances among host species. (PDF)

S4 Table Summary of 16S rRNA amplicon sequencing results for twelve host species. (PDF)

S5 Table Pairwise test results of alpha diversity (Hill_1_) among host species and dietary guilds. Shown are difference in means, upper and lower boundaries and Benjamini-Hochberg adjusted p-values.
(PDF)

S6 Table Pairwise test results of the effect of host species, diet and locality on the microbiota composition based on unweighted UniFrac distances. (PDF)

## References

[1] Schluter J, Peled JU, Taylor BP, Markey KA, Smith M, Taur Y, et al. The gut microbiota is associated with immune cell dynamics in humans. Nature. 2020;588: 303–307. doi: 10.1038/s41586-020-2971-8 33239790 PMC7725892

[2] Dieterich W, Schink M, Zopf Y. Microbiota of the gastrointestinal tract. Med Sci. 2018;6: 116. 10.3390/medsci6040116 PMC6313343 30558253

[3] Ren T, Boutin S, Humphries MM, Dantzer B, Gorrell JC, Coltman DW, et al. Seasonal, spatial, and maternal effects on gut microbiome in wild red squirrels. Microbiome. 2017;5: 163. doi: 10.1186/s40168-017-0382-3 29268780 PMC5740981

[4] Sommer F, Stahlman M, Ilkayeva O, Arnemo JM, Kindberg J, Josefsson J, et al. The gut microbiota modulates energy metabolism in the hibernating brown bear Ursus arctos. Cell Rep. 2016;14: 1655–61. doi: 10.1016/j.celrep.2016.01.026 26854221

[5] Flint HJ, Bayer EA. Plant cell wall breakdown by anaerobic microorganisms from the mammalian digestive tract. Ann NY Acad Sci. 2008;1125: 280–288. doi: 10.1196/annals.1419.022 18378598

[6] Kang W, Kim PS, Tak EJ, Sung H, Sin NR, Hyun DW, et al. Host phylogeny, habitat, and diet are main drivers of the cephalopod and mollusk gut microbiome. Anim microbiome. 2022;4: 30. doi: 10.1186/s42523-022-00184-x 35527289 PMC9082898

[7] Rojas CA, Ramírez-Barahona S, Holekamp KE, Theis KR. Host phylogeny and host ecology structure the mammalian gut microbiota at different taxonomic scales. Anim microbiome. 2021;3: 33. doi: 10.1186/s42523-021-00094-4 33892813 PMC8063394

[8] Waite DW, Taylor MW. Exploring the avian gut microbiota: current trends and future directions. Front Microbiol. 2015;6: 673. doi: 10.3389/fmicb.2015.00673 26191057 PMC4490257

[9] Phillips CD, Phelan G, Dowd SE, McDonough MM, Ferguson AW, Hanson JD, et al. Microbiome analysis among bats describes influences of host phylogeny, life history, physiology, and geography. Mol Ecol. 2012;21: 2617–27. doi: 10.1111/j.1365-294X.2012.05568.x 22519571

[10] Lim SJ, Bordenstein SR. An introduction to phylosymbiosis. Proc R Soc B Biol Sci. 2020;287: 1–10. doi: 10.1098/rspb.2019.2900 32126958 PMC7126058

[11] Kohl KD. Ecological and evolutionary mechanisms underlying patterns of phylosymbiosis in host-associated microbial communities. Philos Trans R Soc B Biol Sci. 2020;375: 20190251. doi: 10.1098/rstb.2019.0251 32200746 PMC7133527

[12] Amato KR, Mallott EK, McDonald D, Dominy NJ, Goldberg T, Lambert JE, et al. Convergence of human and Old World monkey gut microbiomes demonstrates the importance of human ecology over phylogeny. Genome Biol. 2019a;20: 201. doi: 10.1186/s13059-019-1807-z 31590679 PMC6781418

[13] Ross AA, Müller KM, Weese JS, Neufeld JD. Comprehensive skin microbiome analysis reveals the uniqueness of human skin and evidence for phylosymbiosis within the class Mammalia. Proc Natl Acad Sci USA. 2018;115: E5786–E5795. doi: 10.1073/pnas.1801302115 29871947 PMC6016819

[14] Nishida AH, Ochman H. Rates of gut microbiome divergence in mammals. Mol Ecol. 2018;27: 1884–97. doi: 10.1111/mec.14473 29290090 PMC5935551

[15] Ley RE, Hamady M, Lozupone C, Turnbaugh PJ, Ramey RR, Bircher JS, et al. Evolution of mammals and their gut microbes. Science. 2008;320: 1647–51. doi: 10.1126/science.1155725 18497261 PMC2649005

[16] Li H, Zhou R, Zhu J, Huang X, Qu J. Environmental filtering increases with elevation for the assembly of gut microbiota in wild pikas. Microb Biotechnol. 2019;12: 976–992. doi: 10.1111/1751-7915.13450 31380612 PMC6680628

[17] Goertz S, de Menezes AB, Birtles RJ, Fenn J, Lowe AE, MacColl ADC, et al. Geographical location influences the composition of the gut microbiota in wild house mice (Mus musculus domesticus) at a fine spatial scale. PLoS One. 2019;14: e0222501. doi: 10.1371/journal.pone.0222501 31557179 PMC6767902

[18] Amato KR, Yeoman CJ, Kent A, Righini N, Carbonero F, Estrada A, et al. Habitat degradation impacts black howler monkey (Alouatta pigra) gastrointestinal microbiomes. ISME J. 2013;7: 1344–1353. doi: 10.1038/ismej.2013.16 23486247 PMC3695285

[19] Knowles SCL, Eccles RM, Baltrūnaitė L. Species identity dominates over environment in shaping the microbiota of small mammals. Ecol Lett. 2019;22: 826–37. doi: 10.1111/ele.13240 30868708

[20] Delsuc F, Metcalf JL, Parfrey LW, Song SJ, González A, Knight R, et al. Convergence of gut microbiomes in myrmecophagous mammals. Mol Ecol. 2014;23: 1301–1317. doi: 10.1111/mec.12501 24118574

[21] Amato KR, Sanders J, Song SJ, Nute M, Metcalf JL, Thompson LR, et al. Evolutionary trends in host physiology outweigh dietary niche in structuring primate gut microbiomes. ISME J. 2019b;13: 576–87. doi: 10.1038/s41396-018-0175-0 29995839 PMC6461848

[22] Neha SA, Salazar-Bravo J. Fine-scale spatial variation shape fecal microbiome diversity and composition in black-tailed prairie dogs (Cynomys ludovicianus). BMC Microbiol. 2023;23: 5. 36858951 10.1186/s12866-023-02778-0 PMC9979494

[23] Weldon L, Abolins S, Lenzi L, Bourne C, Riley EM, Viney M. The gut microbiota of wild mice. PLoS One. 2015;10: e0134643. doi: 10.1371/journal.pone.0134643 26258484 PMC4530874

[24] Linnenbrink M, Wang J, Hardouin EA, Künzel S, Metzler D, Baines JF. The role of biogeography in shaping diversity of the intestinal microbiota in house mice. Mol Ecol. 2013;22: 1904–16. doi: 10.1111/mec.12206 23398547

[25] Shin JH, Sim M, Lee JY, Shin DM. Lifestyle and geographic insights into the distinct gut microbiota in elderly women from two different geographic locations. J Physiol Anthropol. 2016;35: 31. doi: 10.1186/s40101-016-0121-7 27955701 PMC5151137

[26] Suzuki TA, Martins FM, Nachman MW. Altitudinal variation of the gut microbiota in wild house mice. Mol Ecol. 2018;28: 2378–2390. doi: 10.1111/mec.14905 30346069 PMC6476712

[27] Zouache K, Raharimalala FN, Raquin V, Tran-Van V, Raveloson LHR, Ravelonandro P, et al. Bacterial diversity of field-caught mosquitoes, Aedes albopictus and Aedes aegypti, from different geographic regions of Madagascar. FEMS Microbiol Ecol. 2011;75: 377–389. doi: 10.1111/j.1574-6941.2010.01012.x 21175696

[28] Couch CE, Arnold HK, Crowhurst RS, Jolles AE, Sharpton TJ, Witczak MF, et al. Bighorn sheep gut microbiomes associate with genetic and spatial structure across a metapopulation. Sci Rep. 2020;10: 6582. doi: 10.1038/s41598-020-63401-0 32313214 PMC7171152

[29] Lutz HL, Jackson EW, Webala PW, Babyesiza WS, Kerbis Peterhans JC, Demos TC, et al. Ecology and host identity outweigh evolutionary history in shaping the bat microbiome. mSystems. 2019;4: e00511–e00519. doi: 10.1128/mSystems.00511-19 31719140 PMC7407897

[30] Wang Z, Zhang C, Li G, Yi X. The influence of species identity and geographic locations on gut microbiota of small rodents. Front Microbiol. 2022;13: 983660. doi: 10.3389/fmicb.2022.983660 36532505 PMC9751661

[31] Weinstein SB, Martinez-Motaa R, Stapletona TE, Klure DM, Greenhalgh R, Orr TJ, et al. Microbiome stability and structure is governed by host phylogeny over diet and geography in woodrats (Neotoma spp.). PNAS. 2021;118: e2108787118. doi: 10.1073/pnas.2108787118 34799446 PMC8617456

[32] Sikes RS, Gannon WL, Animal Care and Use Committee of the American Society of Mammalogists. Guidelines of the American Society of Mammalogists for the use of wild mammals in research. J Mamm. 2011;92: 235–253. 10.1093/jmammal/gyw078 PMC5909806 29692469

[33] Environmental Protection Agency U.S. Level III ecoregions of the continental United States (revision of Omernik, 1987): Corvallis, Oregon, U.S. Environmental Protection Agency-National Health and Environmental Effects Research Laboratory. 2003; Map M-1, various scales.

[34] Phillips CD, Hanson JD, Wilkinson JE, Koenig L, Rees E, Webala P, et al. Microbiome Structural and Functional Interactions across Host Dietary Niche Space. Integr Comp Biol. 2017;57: 743–755. doi: 10.1093/icb/icx011 28662574

[35] Zhang J, Kobert K, Flouri T, Stamatakis A. PEAR: a fast and accurate Illumina Paired-End reAd mergeR. Bioinformatics. 2014;30: 614–20. doi: 10.1093/bioinformatics/btt593 24142950 PMC3933873

[36] Edgar RC. Search and clustering orders of magnitude faster than BLAST. Bioinformatics. 2010;26: 2460–2461. doi: 10.1093/bioinformatics/btq461 20709691

[37] Yilmaz P, Parfrey LW, Yarza P, Gerken J, Pruesse E, Quast C, et al. The SILVA and "All-species Living Tree Project (LTP)" taxonomic frameworks. Nucleic Acids Res. 2014;42: D643–D648. doi: 10.1093/nar/gkt1209 24293649 PMC3965112

[38] Nawrocki EP, Eddy SR. ssu-align: a tool for structural alignment of SSU rRNA sequences; 2010. Available from: http://selab. janelia. org/software.

[39] Price MN, Dehal PS, Arkin AP. FastTree 2 ‐ approximately maximum-likelihood trees for large alignments. PLoS One. 2010;5: e9490. doi: 10.1371/journal.pone.0009490 20224823 PMC2835736

[40] McMurdie PJ, Holmes S. phyloseq: an R package for reproducible interactive analysis and graphics of microbiome census data. PLoS One. 2013;8: e61217. doi: 10.1371/journal.pone.0061217 23630581 PMC3632530

[41] R Core Team. R: A language and environment for statistical computing. R Foundation for Statistical Computing, Vienna, Austria; 2022. Available from: https://www.R-project.org/.

[42] Revell LJ. phytools: an R package for phylogenetic comparative biology (and other things). Methods Ecol Evol. 2012;3: 217–23.

[43] Paradis E, Claude J, Strimmer K. APE: analyses of phylogenetics and evolution in R language. Bioinformatics. 2004;20: 289–90. doi: 10.1093/bioinformatics/btg412 14734327

[44] Oksanen J, Blanchet FG, Kindt R, Legendre P, Minchin PR, O’Hara RB, et al. vegan: Community Ecology Package. R package version 2.3–4; 2016. Available from: https://CRAN.R-project.org/ package=vegan.

[45] Kembel SW, Cowan PD, Helmus MR, Cornwell WK, Morlon H, Ackerly DD, et al. Picante: R tools for integrating phylogenies and ecology. Bioinformatics. 2010;26: 1463–4. doi: 10.1093/bioinformatics/btq166 20395285

[46] Wickham H, Seidel D. scales: Scale Functions for Visualization. R package version 1.1.1; 2020. Available from: https://CRAN.R-project.org/package=scales.

[47] Wickham H. Reshaping Data with the reshape Package. J Stat Softw. 2007;21: 1–20. Available from: http://www.jstatsoft.org/v21/i12/.

[48] Pedro MA. pairwiseAdonis: Pairwise multilevel comparison using adonis. R package version 0.4; 2020.

[49] Bates D, Maechler M, Bolker B, Walker S. Fitting linear mixedeffects models using lme4. J Stat Softw. 2015;67: 1–48.

[50] Wickham H. ggplot2: Elegant Graphics for Data Analysis. Springer-Verlag New York; 2016.

[51] Beule L, Karlovsky P. Improved normalization of species count data in ecology by scaling with ranked subsampling (SRS): application to microbial communities. PeerJ. 2020; 8:e9593. doi: 10.7717/peerj.9593 32832266 PMC7409812

[52] Bokulich N, Subramanian S, Faith J, Gevers D, Gordon JI, Knight R, et al. Quality-filtering vastly improves diversity estimates from Illumina amplicon sequencing. Nat Methods. 2013;10: 57–59. doi: 10.1038/nmeth.2276 23202435 PMC3531572

[53] Faith DP. Conservation evaluation and phylogenetic diversity. Biol Conserv. 1992;61: 1–10.

[54] Garland T Jr, Dickerman AW, Janis CM, Jones JA. Phylogenetic analysis of covariance by computer simulation. Syst Biol. 1993;42: 265–292.

[55] Lozupone C, Knight R. UniFrac: a new phylogenetic method for comparing microbial communities. Appl Environ Microbiol. 2005;71: 8228–35. doi: 10.1128/AEM.71.12.8228-8235.2005 16332807 PMC1317376

[56] Cao Y, Dong Q, Wang D, Zhang P, Liu Y, Niu C. microbiomeMarker: an R/Bioconductor package for microbiome marker identification and visualization. Bioinformatics. 2022;38: 4027–4029. doi: 10.1093/bioinformatics/btac438 35771644

[57] Segata N, Izard J, Waldron L, Gevers D, Miropolsky L, Garrett WS, et al. Metagenomic biomarker discovery and explanation. Genome Biol. 2011;12: R60. doi: 10.1186/gb-2011-12-6-r60 21702898 PMC3218848

[58] Edgar RC. MUSCLE: multiple sequence alignment with high accuracy and high throughput. Nucleic Acids Res. 2004;32: 1792–1797. doi: 10.1093/nar/gkh340 15034147 PMC390337

[59] Stamatakis A. RAxML version 8: a tool for phylogenetic analysis and post-analysis of large phylogenies. Bioinformatics. 2014;30: 1312–1313. doi: 10.1093/bioinformatics/btu033 24451623 PMC3998144

[60] Darriba D, Taboada GL, Doallo R, Posada D. jModelTest 2: more models, new heuristics and parallel computing. Nat Methods. 2012;9: 772. doi: 10.1038/nmeth.2109 22847109 PMC4594756

[61] Burnham KP, Anderson DR. Multimodel inference: understanding AIC and BIC in model selection. Sociol Methods Res. 2004;33: 261–304.

[62] Hasegawa M, Kishino H, Yano TA. Dating of the humanape splitting by a molecular clock of mitochondrial DNA. J Mol Evol. 1985;22: 160–174. doi: 10.1007/BF02101694 3934395

[63] Felsenstein J. Confidence limits on phylogenies: an approach using the bootstrap. Evolution. 1985;39: 783–791. doi: 10.1111/j.1558-5646.1985.tb00420.x 28561359

[64] Ronquist F, Teslenko M, Van Der Mark P, Ayres DL, Darling A, Höhna S, et al. MrBayes 3.2: efficient Bayesian phylogenetic inference and model choice across a large model space. Syst Biol. 2012;61: 539–542. doi: 10.1093/sysbio/sys029 22357727 PMC3329765

[65] Huelsenbeck JP, Larget B, Miller RE, Ronquist F. Potential applications and pitfalls of Bayesian inference of phylogeny. Syst Biol. 2002;51: 673–688. doi: 10.1080/10635150290102366 12396583

[66] Li J, Zhan S, Liu X, Lin Q, Jiang J, Li X. Divergence of fecal microbiota and their associations with host phylogeny in Cervinae. Front Microbiol. 2018;9: 1–11. 30214431 10.3389/fmicb.2018.01823 PMC6125396

[67] Henderson G, Cox F, Ganesh S, Jonker A, Young W, Janssen PH, et al. Rumen microbial community composition varies with diet and host, but a core microbiome is found across a wide geographical range. Sci Rep. 2015;5: 14567. doi: 10.1038/srep14567 26449758 PMC4598811

[68] Greene LK, McKenney EA, O’Connell TM, Drea CM. The critical role of dietary foliage in maintaining the gut microbiome and metabolome of folivorous sifakas. Sci Rep. 2018;8: 1–13. 30262842 10.1038/s41598-018-32759-7 PMC6160417

[69] Teng Y, Yang X, Li G, Zhu Y, Zhang Z. Habitats Show More Impacts Than Host Species in Shaping Gut Microbiota of Sympatric Rodent Species in a Fragmented Forest. Front Microbiol. 2022;13: 811990. doi: 10.3389/fmicb.2022.811990 35197954 PMC8859092

[70] Jovel J, Dieleman LA, Kao D, Mason AL, Wine E. The human gut microbiome in health and disease. Metagenomics. 2018;197–213.

[71] Kohl KD, Denise DM. The woodrat gut microbiota as an experimental system for understanding microbial metabolism of dietary toxins. Front Microbiol. 2016;7: 1–9. 27516760 10.3389/fmicb.2016.01165 PMC4963388

[72] Wang J, Lang T, Shen J, Dai J, Tian L, Wang X. Core Gut Bacteria Analysis of Healthy Mice. Front Microbiol. 2019;10: 887. doi: 10.3389/fmicb.2019.00887 31105675 PMC6491893

[73] Maier L, Prteanu M, Kuhn M, Zeller G, Telzerow A, Anderson EE, et al. Extensive impact on non-antibiotic drugs on human gut bacteria. Nature. 2018;555: 623–628. 29555994 10.1038/nature25979 PMC6108420

[74] Cheng J, Ringel-Kulka T, Heikamp-de Jong I, Ringel Y, Carroll I, de Vos WM, et al. Discordant temporal development of bacterial phyla and the emergence of core in the fecal microbiota of young children. ISME J. 2016;10: 1002–1014. doi: 10.1038/ismej.2015.177 26430856 PMC4796939

[75] Kohl KD, Dieppa-Colón E, Goyco-Blas J, Peralta-Martínez K, Scafidi L, Shah S, et al. Gut Microbial Ecology of Five Species of Sympatric Desert Rodents in Relation to Herbivorous and Insectivorous Feeding Strategies. Integr Comp Biol. 2022;6: 237–251. doi: 10.1093/icb/icac045 35587374

[76] Donohoe DR, Garge N, Zhang X, Sun W, O’Connell TM, Bunger MK, et al. The microbiome and butyrate regulate energy metabolism and autophagy in the mammalian colon. Cell Metab. 2011;13: 517–526. doi: 10.1016/j.cmet.2011.02.018 21531334 PMC3099420

[77] Sharma B, Singh N. Attenuation of vascular dementia by sodium butyrate in streptozotocin diabetic rats. Psychopharm. 2011;215: 677–687. doi: 10.1007/s00213-011-2164-0 21225418

[78] Karasov WH, Martínez del Rio C. Physiological ecology: how animals process energy, nutrients, and toxins. Princeton (NJ): Princeton University Press; 2007.

[79] Greene LK, Clayton JB, Rothman RS, Semel BP, Semel MA, Gillespie TR, et al. Local habitat, not phylogenetic relatedness, predicts gut microbiota better within folivorous than frugivorous lemur lineages. Biol Lett. 2019;15: 5–11. 10.1098/rsbl.2019.0028 PMC6597504 31185820

[80] Schmidt E, Mykytczuk N, Schulte-Hostedde AI. Effects of the captive and wild environment on diversity of the gut microbiome of deer mice (Peromyscus maniculatus). ISME J. 2019;13: 1293–1305. doi: 10.1038/s41396-019-0345-8 30664674 PMC6474230

[81] Groussin M, Mazel F, Alm EJ. Co-evolution and co-speciation of host-gut Bacteria systems. Cell Host Microbe. 2020;28: 12–22. doi: 10.1016/j.chom.2020.06.013 32645351

[82] Gerardo NM, Hoang KL, Stoy KS. Evolution of animal Immunity in the light of beneficial symbioses. Philos Trans R Soc B2. 2020;375: 20190601. doi: 10.1098/rstb.2019.0601 32772666 PMC7435162

[83] Raulo A, Ruokolainen L, Lane A, Amato K, Knight R, Leigh S, et al. Social behaviour and gut microbiota in red-bellied lemurs (Eulemur rubriventer): in search of the role of immunity in the evolution of sociality. J Anim Ecol. 2017;87: 1–12. doi: 10.1111/1365-2656.12781 29205327

[84] Goertz S, de Menezes AB, Birtles RJ, Fenn J, Lowe AE, MacColl ADC, et al. Geographical location influences the composition of the gut microbiota in wild house mice (Mus musculus domesticus) at a fine spatial scale. PLoS One. 2019;14:e0222501. doi: 10.1371/journal.pone.0222501 31557179 PMC6767902

[85] Linnenbrink M, Wang J, Hardouin EA, Künzel S, Metzler D, Baines JF. The role of biogeography in shaping diversity of the intestinal microbiota in house mice. Mol Ecol. 2013; doi: 10.1111/mec.12206 . 23398547

[86] Kylie J, Weese JS, Turner PV. Comparison of the fecal microbiota of domestic commercial meat, laboratory, companion, and shelter rabbits (Oryctolagus cuniculi). BMC Vet Res. 2018;14:143. doi: 10.1186/s12917-018-1464-6 29703196 PMC5924505

[87] Crowley EJ, King JM, Wilkinson T, Worgan HJ, Huson KM, Rose MT, McEwan NR. Comparison of the microbial population in rabbits and guinea pigs by next generation sequencing. PLoS ONE. 2017;12:1–14. doi: 10.1371/journal.pone.0165779 28182658 PMC5300138

