# Supplementary figures and images for: Impacts of host phylogeny, diet, and geography on the gut microbiome of rodents
Source: PLoS One. 2025 Jan 16;20(1):e0316101. doi: 10.1371/journal.pone.0316101 (PMC11737772; doi:10.1371/journal.pone.0316101)

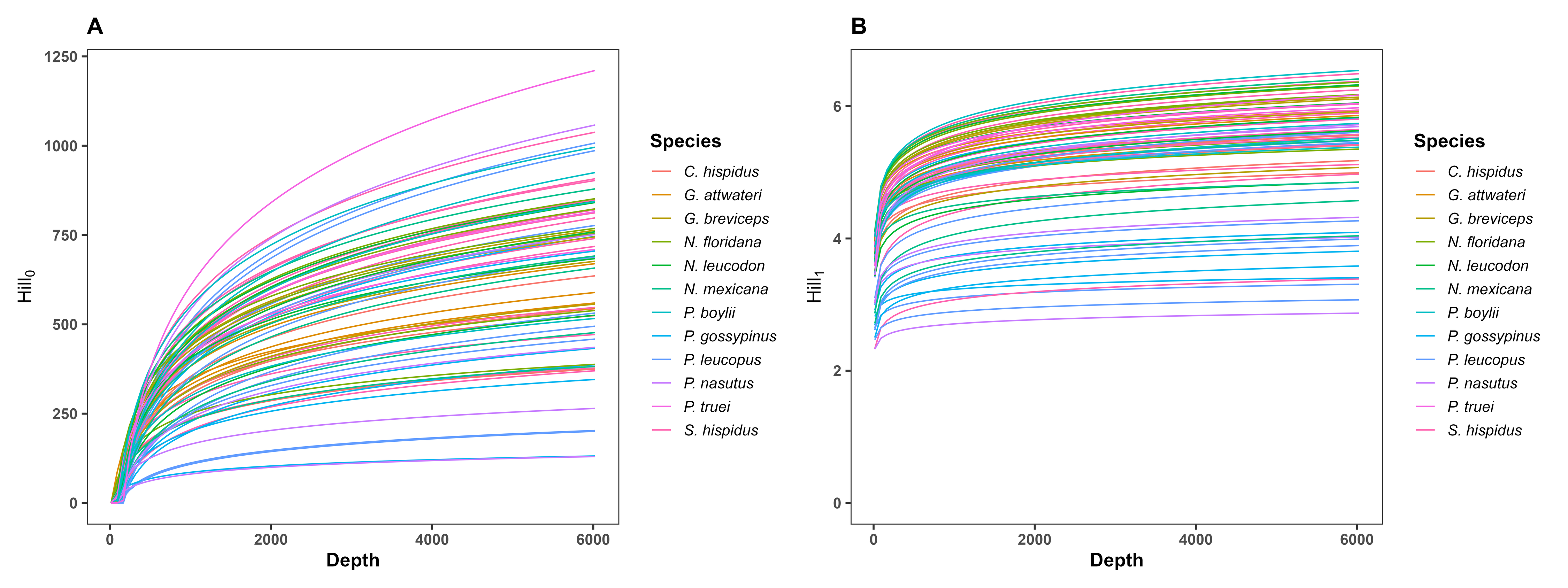

Supplement: S1 Fig — Rarefaction curves of Hill0 (A) and Hill1 (B) estimates for host species with subsampling between 500 and 6,000 reads at a step size of 500. (TIF) [file pone.0316101.s001.tif]
