# Supplementary material for: Impacts of host phylogeny, diet, and geography on the gut microbiome of rodents
Source: PLoS One. 2025 Jan 16;20(1):e0316101. doi: 10.1371/journal.pone.0316101 (PMC11737772; doi:10.1371/journal.pone.0316101)
Supplement: S1 Table — GenBank accession numbers and museum voucher numbers used in this study are listed. (PDF) [file pone.0316101.s002.pdf]

S1 Table. Cytochrome-b sequences of twelve species of hosts including one outgroup species obtained from GenBank. GenBank accession numbers and museum voucher numbers used in this study are listed.

GenBank Accession #	Museum voucher #	Organism
AY926391	LVT1099	*Chaetodipus hispidus*
AF172832	-	*Chaetodipus hispidus*
AY393936	LSUMZ29596	*Geomys attwateri*
AY393937	AK5455	*Geomys attwateri*
AY393938	AK7920	*Geomys attwateri*
U65297	-	*Geomys breviceps*
U65298	-	*Geomys breviceps*
KC680049	LSUMZ29336	*Geomys breviceps*
AY926386	LVT5500	*Geomys breviceps*
AF294344	TK52115	*Neotoma floridana*
AF294343	TK51632	*Neotoma floridana*
AF294342	TK29708	*Neotoma floridana*
AF294341	TK27751	*Neotoma floridana*
AF294340	TK25365	*Neotoma floridana*
AF294339	TK23385	*Neotoma floridana*
AF294338	SP799	*Neotoma floridana*
AF294337	SP798	*Neotoma floridana*
AF294336	NK64158	*Neotoma floridana*
AF294335	NK64089	*Neotoma floridana*
AF294334	NK52064	*Neotoma floridana*
AF294333	-	*Neotoma floridana*
KY754059	-	*Neotoma floridana*
DQ179854	NK64089	*Neotoma floridana*
DQ179821	TK28244	*Neotoma floridana*
DQ179820	TK27751	*Neotoma floridana*
DQ179819	TK52109	*Neotoma floridana*
MZ099558	-	*Neotoma leucodon*
MW419110	MZFC12332	*Neotoma leucodon*
MK253565	TK185524	*Neotoma leucodon*
MK253564	TK181176	*Neotoma leucodon*
MK253561	TK185560	*Neotoma leucodon*
MK253560	TK181271	*Neotoma leucodon*
MK253559	TK163106	*Neotoma leucodon*
DQ179839	TK48594	*Neotoma leucodon*
DQ179815	TK49716	*Neotoma leucodon*
GU220381	Tk133448	*Neotoma leucodon*
KM488350	TTU111790	*Neotoma leucodon*
KM488349	TTU111780	*Neotoma leucodon*
KM488348	MSB58295	*Neotoma leucodon*
KF733992	TK92410	*Neotoma leucodon*
MW419114	MZFC11989	*Neotoma Mexicana*
MW419113	MZFC12327	*Neotoma Mexicana*

MZ064563	TK182740	*Neotoma Mexicana*
MK803421	CMC1204	*Neotoma Mexicana*
KY754062	-	*Neotoma Mexicana*
DQ179847	TK78350	*Neotoma Mexicana*
DQ179846	TK51346	*Neotoma Mexicana*
DQ179845	TK45631	*Neotoma Mexicana*
AF294346	TK90038	*Neotoma Mexicana*
AF294345	TK78350	*Neotoma Mexicana*
KM488363	TTU110064	*Neotoma Mexicana*
FJ716223	-	*Neotoma Mexicana*
FJ716222	-	*Neotoma Mexicana*
OK205192	-	*Peromyscus boylii*
EF989971	CMNH103724	*Peromyscus boylii*
EF989979	ASNHC3449	*Peromyscus boylii*
DQ000478	TK24389	*Peromyscus boylii*
DQ861379	BYU19433	*Peromyscus boylii*
MQ684862	TK83719	*Peromyscus boylii*
MQ684861	TK72944	*Peromyscus boylii*
MQ684860	TK148269	*Peromyscus boylii*
AY322506	TK48617	*Peromyscus boylii*
AY322505	TK48590	*Peromyscus boylii*
AY322504	TK93089	*Peromyscus boylii*
MN124380	TK24235	*Peromyscus gosyypinus*
DQ385625	MSB53305	*Peromyscus gosyypinus*
DQ385624	MSB53304	*Peromyscus gosyypinus*
FJ214686	TK90655	*Peromyscus gosyypinus*
DQ973102	TTU80682	*Peromyscus gosyypinus*
DQ973101	TTU55019	*Peromyscus gosyypinus*
MN124383	TK167003	*Peromyscus leucopus*
MK410341	-	*Peromyscus leucopus*
KY064168	TTU57149	*Peromyscus leucopus*
KY754106	-	*Peromyscus leucopus*
EF989980	CMNH92801	*Peromyscus leucopus*
DQ000483	TK121168	*Peromyscus leucopus*
EF989979	ROM101861	*Peromyscus leucopus*
AY859474	BYU15919	*Peromyscus leucopus*
DQ973104	TTU104424	*Peromyscus leucopus*
DQ861376	BYU15919	*Peromyscus leucopus*
KJ810666	TK148990	*Peromyscus leucopus*
AY263615	-	*Peromyscus leucopus*
OL631916	CRD12272	*Peromyscus nasutus*
MN022895	TTUM138362	*Peromyscus nasutus*
MN022894	TTUM136503	*Peromyscus nasutus*
MN022893	TTUM36097	*Peromyscus nasutus*
AY376426	TK83576	*Peromyscus nasutus*
OL631929	MSB304293	*Peromyscus truei*

OL631928	LSUMZ31403	*Peromyscus truei*
MN022915	TTUM47286	*Peromyscus truei*
MN022914	TTUM69561	*Peromyscus truei*
MN022913	TTUM47300	*Peromyscus truei*
MN022912	TTUM36056	*Peromyscus truei*
MN022911	TTUM36064	*Peromyscus truei*
MN022910	TTUM36068	*Peromyscus truei*
MN022909	TTUM36060	*Peromyscus truei*
MN022908	TTUM61541	*Peromyscus truei*
MN022907	TTUM120632	*Peromyscus truei*
MN022906	TTUM120631	*Peromyscus truei*
MN022905	TTUM120630	*Peromyscus truei*
MN022904	TTUM136502	*Peromyscus truei*
MN022902	TTUM142742	*Peromyscus truei*
MN022901	TTUM142741	*Peromyscus truei*
MN022899	TTUM119068	*Peromyscus truei*
MN022898	TTUM119069	*Peromyscus truei*
MN022897	TTUM100425	*Peromyscus truei*
MN022896	TTUM55604	*Peromyscus truei*
EU073178	TK137308	*Sigmodon hispidus*
EU073177	TK137315	*Sigmodon hispidus*
AF425227	TK90616	*Sigmodon hispidus*
AF425214	ASK4996	*Sigmodon hispidus*
AF425213	OK5830	*Sigmodon hispidus*
AF425212	TK77280	*Sigmodon hispidus*
AF425211	TK24993	*Sigmodon hispidus*
AF425210	TK52127	*Sigmodon hispidus*
AF425209	OK5840	*Sigmodon hispidus*
AF425208	TK92509	*Sigmodon hispidus*
AF425207	TK92507	*Sigmodon hispidus*
AF425206	TK51918	*Sigmodon hispidus*
AF425205	M154	*Sigmodon hispidus*
AF425204	M1915	*Sigmodon hispidus*
AF425203	TK28256	*Sigmodon hispidus*
AF425202	TK77942	*Sigmodon hispidus*
AF425201	TK90087	*Sigmodon hispidus*
AF425200	TK84530	*Sigmodon hispidus*
AF425199	TK32481	*Sigmodon hispidus*
AF435110	TK47553	*Sigmodon hispidus*
-	MSB45953	*Sylvilagus holzneri* (outgroup)
