# Supplementary material for: Impacts of host phylogeny, diet, and geography on the gut microbiome of rodents
Source: PLoS One. 2025 Jan 16;20(1):e0316101. doi: 10.1371/journal.pone.0316101 (PMC11737772; doi:10.1371/journal.pone.0316101)
Supplement: S2 Table — (PDF) [file pone.0316101.s003.pdf]

S2 Table. Dietary profiles for the twelve rodent species included in this study.

**Host species**	**Dietary guild**	**% Plants consumed**	**Data reference**
C. hispidus	Granivore	10	Hamish *et al*. 2014
G. attwateri	Herbivore	60	Williams and Cameron 1986
G. breviceps	Herbivore	60	Hamish *et al*. 2014
N. floridana	Herbivore	87	Wagle and Feldhamer 1997
N. leucodon	Herbivore	82	Dial 1998
N. mexicana	Herbivore	68	Justice 1985
P. leucopus	Omnivore	5	Hope and Robert 2007
P. nasutus	Omnivore	20	Hamish *et al*. 2014
P. gossypinus	Omnivore	50	Schmidly and Bradley 2016
P. boylii	Omnivore	27	Hope and Robert 2007
P. truei	Herbivore	6	Hope and Robert 2007
S. hispidus	Herbivore	50	Hamish *et al*. 2014
