# Supplementary material for: Impacts of host phylogeny, diet, and geography on the gut microbiome of rodents
Source: PLoS One. 2025 Jan 16;20(1):e0316101. doi: 10.1371/journal.pone.0316101 (PMC11737772; doi:10.1371/journal.pone.0316101)
Supplement: S3 Table — (PDF) [file pone.0316101.s004.pdf]

S3 Table. Pairwise genetic distances among host species.

	C_hispidus	***G.attwateri***	***G.breviceps***	***S.hispidus***	***P.leucopus***	***P.gossypinus***	***P.nasutus***	***P.boylii***	***P.truei***	***N.mexicana***	***N.leucodon***	***N.floridana***
C_hispidus												
G.attwateri	0.2978											
G.breviceps	0.2986	0.1391										
S.hispidus	0.3674	0.3469	0.3570									
P.leucopus	0.3292	0.3151	0.3232	0.2527								
P.gossypinus	0.3151	0.3173	0.3175	0.2628	0.0539							
P.nasutus	0.3244	0.3241	0.3335	0.2412	0.1504	0.1708						
P.boylii	0.3255	0.3326	0.3227	0.2364	0.1295	0.1424	0.1278					
P.truei	0.3305	0.3287	0.3306	0.2493	0.1697	0.1736	0.1347	0.1457				
N.mexicana	0.3460	0.3301	0.3432	0.2559	0.1793	0.1742	0.1951	0.1941	0.2078			
N.leucodon	0.3287	0.3231	0.3094	0.2557	0.1930	0.1843	0.2058	0.2108	0.2119	0.1208		
N.floridana	0.3410	0.3133	0.3157	0.2691	0.1920	0.1971	0.2120	0.2119	0.2257	0.1337	0.1211	
