# Supplementary material for: Impacts of host phylogeny, diet, and geography on the gut microbiome of rodents
Source: PLoS One. 2025 Jan 16;20(1):e0316101. doi: 10.1371/journal.pone.0316101 (PMC11737772; doi:10.1371/journal.pone.0316101)
Supplement: S4 Table — (PDF) [file pone.0316101.s005.pdf]

S4 Table. Summary of 16S rRNA amplicon sequencing results for twelve host species.

**Host species**	**Sample ID**	**Reads**	**Filtered reads**	**$Hill_0$**	**$Hill_1$**	**Faith's PD**
C. hispidus	TK181435	15742	13761	628	5.35	13.45
	TK181454	22356	19235	686	5.10	14.46
	TK182846	18359	16121	558	5.48	13.41
	TK182851	9799	8499	459	4.92	14.99
G. attwateri	TK181440	34473	29032	543	5.81	10.13
	TK181451	15286	13074	656	5.58	15.19
	TK181452	12603	10908	644	5.44	13.92
	TK182850	15282	13093	749	5.78	15.64
G. breviceps	TK182925	16966	14819	828	6.02	16.82
	TK182926	33732	28920	705	5.80	14.63
	TK182964	21720	18033	617	5.04	12.31
	TK182965	21658	18342	806	6.01	16.67
N. floridana	TK181456	9948	8677	650	5.50	14.12
	TK182844	17401	14854	839	6.09	17.89
	TK182848	13360	11720	942	6.18	20.44
	TK182869	17805	15486	867	6.05	19.28
	TK182881	32771	27905	417	5.24	9.85
	TK182956	15379	13138	821	6.25	16.54
N. leucodon	TK185524	9869	8455	661	4.79	18.09
	TK185529	15375	13199	699	5.93	13.87
	TK185560	13488	11583	961	6.25	18.90
	TK185571	60726	52041	986	5.78	19.71
	TK185572	30716	26265	785	5.45	13.98
N. mexicana	TK185578	18971	16282	799	5.46	16.34
	TK185581	9034	7689	432	4.80	9.06
	TK185590	20997	18042	737	4.48	15.64
	TK185592	16954	14703	539	3.98	12.92
	TK185595	14149	12202	980	6.31	16.48
P. boylii	TK185603	19345	16217	1101	5.44	22.88
	TK185604	21204	17922	564	5.25	14.49
	TK185609	31890	27473	952	5.65	21.38
	TK185610	16225	13745	1110	6.44	20.97
P. gossypinus	TK181476	20389	17245	758	5.70	17.59
	TK181479	15060	12762	915	5.37	21.49
	TK181480	22882	19872	510	4.08	15.72
	TK182857	19372	17256	380	3.75	9.57
	TK182867	12894	11066	151	3.39	7.13
	TK182871	12201	10492	513	3.56	14.98
P. leucopus	TK181433	18870	16126	1198	5.58	23.24
	TK181483	26164	22631	227	3.26	8.28
	TK182840	44529	37597	846	5.41	17.49
	TK182841	17934	15757	528	4.21	13.95
	TK182953	20077	17636	247	3.05	10.28
	TK182954	48606	41142	1111	5.60	22.83
	TK182995	35668	32317	612	3.91	15.79
	TK185525	36625	31603	760	4.69	18.08

	TK185526	23425	20375	605	3.90	17.88
	TK185527	17403	14867	884	5.47	19.77
P. nasutus	TK185541	30239	25924	246	3.89	6.97
	TK185542	9551	8459	897	5.65	19.68
	TK185601	18597	16121	142	2.79	5.84
	TK185605	20440	17977	492	4.28	13.55
	TK185607	19818	17051	1237	5.65	24.36
P. truei	TK185543	21493	18286	913	5.88	19.29
	TK185608	9913	8478	837	5.98	18.82
	TK185621	31777	26711	1367	6.04	24.65
S. hispidus	TK181463	21373	18286	824	5.55	16.84
	TK181465	11054	9643	421	5.07	9.18
	TK181472	29874	26149	948	5.44	14.99
	TK181482	16928	14460	994	6.27	13.84
	TK181486	17684	15194	950	5.72	16.61
	TK181491	16854	15273	496	5.33	11.63
	TK181492	30951	26390	807	5.55	13.10
	TK181493	21361	18295	887	4.85	16.17
	TK181497	10530	8884	1050	6.15	16.98
	TK182845	19673	17187	977	5.85	17.50
	TK182852	16093	13589	432	3.35	9.60
	TK182853	13950	12160	612	5.46	15.70
	TK182855	17569	15025	906	5.93	15.70
	TK182859	17499	15050	965	5.86	19.50
