# Supplementary material for: Impacts of host phylogeny, diet, and geography on the gut microbiome of rodents
Source: PLoS One. 2025 Jan 16;20(1):e0316101. doi: 10.1371/journal.pone.0316101 (PMC11737772; doi:10.1371/journal.pone.0316101)
Supplement: S5 Table — Shown are difference in means, upper and lower boundaries and Benjamini-Hochberg adjusted p-values. (PDF) [file pone.0316101.s006.pdf]

S5 Table. Pairwise test results of alpha diversity ($Hill_1$) among host species and dietary guilds. Shown are difference in means, upper and lower boundaries and Benjamini-Hochberg adjusted p-values.

**Host species pairs**	**Difference**	**Lower**	**Upper**	**P.adj**
C. hispidus-G. attwateri	0.423462238	-1.365051327	2.211975802	0.999602
C. hispidus-G. breviceps	0.499078329	-1.289435236	2.287591894	0.998191
C. hispidus-N. floridana-	0.66956695	-0.96311509	2.302248989	0.959939
C. hispidus-N. leucodon-	0.435007549	-1.261725398	2.131740496	0.999162
C. hispidus-N. mexicana-	-0.211222411	-1.907955359	1.485510536	0.999999
C. hispidus-P. boylii	0.503713953	-1.284799612	2.292227518	0.998036
C. hispidus-P. gossypinus	-0.905015402	-2.537697442	0.727666638	0.762742
C. hispidus-P. leucopus	-0.713151482	-2.209529289	0.783226324	0.894072
C. hispidus-P. nasutus	-0.73336863	-2.430101577	0.963364317	0.942701
C. hispidus-P. truei	0.75632262	-1.175492822	2.688138061	0.971227
C. hispidus-S. hispidus	0.307208057	-1.116131146	1.730547261	0.999836
G. attwateri-G. breviceps	0.075616092	-1.712897473	1.864129657	1
G. attwateri-N. floridana	0.246104712	-1.386577328	1.878786752	0.999996
G. attwateri-N. leucodon	0.011545312	-1.685187636	1.708278259	1
G. attwateri-N. mexicana	-0.634684649	-2.331417596	1.062048298	0.979478
G. attwateri-P. boylii	0.080251715	-1.70826185	1.86876528	1
G. attwateri-P. gossypinus	-1.328477639	-2.961159679	0.3042044	0.220445
G. attwateri-P. leucopus	-1.13661372	-2.632991527	0.359764087	0.31182
G. attwateri-P. nasutus	-1.156830868	-2.853563815	0.53990208	0.474867
G. attwateri-P. truei	0.332860382	-1.598955059	2.264675824	0.999983
G. attwateri-S. hispidus	-0.11625418	-1.539593384	1.307085024	1
G. breviceps-N. floridana	0.17048862	-1.462193419	1.80317066	1
G. breviceps-N. leucodon	-0.06407078	-1.760803728	1.632662167	1
G. breviceps-N. mexicana	-0.710300741	-2.407033688	0.986432207	0.953811
G. breviceps-P. boylii	0.004635623	-1.783877941	1.793149188	1
G. breviceps-P. gossypinus	-1.404093731	-3.036775771	0.228588309	0.158598
G. breviceps-P. leucopus	-1.212229812	-2.708607618	0.284147995	0.225837
G. breviceps-P. nasutus	-1.232446959	-2.929179907	0.464285988	0.377548
G. breviceps-P. truei	0.25724429	-1.674571151	2.189059732	0.999999
G. breviceps-S. hispidus	-0.191870272	-1.615209476	1.231468932	0.999999
N. floridana-N. leucodon	-0.234559401	-1.766150915	1.297032114	0.999995
N. floridana-N. mexicana	-0.880789361	-2.412380875	0.650802153	0.719336
N. floridana-P. boylii	-0.165852997	-1.798535037	1.466829043	1
N. floridana-P. gossypinus	-1.574582351	-3.034897562	-0.114267141	0.024005*
N. floridana-P. leucopus	-1.382718432	-2.688864064	-0.0765728	0.029006*
N. floridana-P. nasutus	-1.40293558	-2.934527094	0.128655934	0.102673
N. floridana-P. truei	0.08675567	-1.701757895	1.875269235	1
N. floridana-S. hispidus	-0.362358892	-1.584146255	0.859428471	0.996905
N. leucodon-N. mexicana	-0.646229961	-2.245925124	0.953465203	0.963967
N. leucodon-P. boylii	0.068706404	-1.628026544	1.765439351	1
N. leucodon-P. gossypinus	-1.340022951	-2.871614465	0.191568563	0.141775
N. leucodon-P. leucopus	-1.148159032	-2.533535682	0.237217619	0.198644
N. leucodon-P. nasutus	-1.168376179	-2.768071343	0.431318985	0.369264
N. leucodon-P. truei	0.321315071	-1.525853796	2.168483938	0.999981
N. leucodon-S. hispidus	-0.127799492	-1.433945124	1.17834614	1
N. Mexicana-P. boylii	0.714936364	-0.981796583	2.411669312	0.951712
N. Mexicana-P. gossypinus	-0.69379299	-2.225384505	0.837798524	0.922368
N. Mexicana-P. leucopus	-0.501929071	-1.887305721	0.883447579	0.98392
N. Mexicana-P. nasutus	-0.522146219	-2.121841382	1.077548945	0.993024
N. Mexicana-P. truei	0.967545031	-0.879623836	2.814713898	0.820809

N. Mexicana-S. hispidus	0.518430469	-0.787715163	1.824576101	0.968225
P. boylii-P. gossypinus	-1.408729355	-3.041411394	0.223952685	0.155286
P. boylii-P. leucopus	-1.216865435	-2.713243242	0.279512371	0.221151
P. boylii-P. nasutus	-1.237082583	-2.93381553	0.459650365	0.371894
P. boylii-P. truei	0.252608667	-1.679206775	2.184424108	0.999999
P. boylii-S. hispidus	-0.196505895	-1.619845099	1.226833308	0.999998
P. gossypinus-P. leucopus	0.191863919	-1.114281712	1.498009551	0.999997
P. gossypinus-P. nasutus	0.171646772	-1.359944742	1.703238286	1
P. gossypinus-P. truei	-1.061338022	-2.042717553	0.14985158	0.124084
P. gossypinus-S. hispidus	1.212223459	-0.009563904	2.434010822	0.053636*
P. leucopus-P. nasutus	-0.020217148	-1.405593798	1.365159503	1
P. leucopus-P. truei	1.469474102	-0.195541414	3.134489618	0.133727
P. leucopus-S. hispidus	1.02035954	-0.012239248	2.052958328	0.055594*
P. nasutus-P. truei	1.48969125	-0.357477617	3.336860117	0.231425
P. nasutus-S. hispidus	1.040576687	-0.265568944	2.346722319	0.246944
P. truei-S. hispidus	-0.449114562	-2.048809726	1.150580602	0.99809
**Dietary guild**				
Granivore -Herbivore	0.373895	-0.60041	1.348199	0.629923
Granivore-Omnivore	-0.56854	-1.57124	0.434152	0.368258
Herbivore-Omnivore	-0.94244	-1.41278	-0.4721	0.000026*
