# Supplementary material for: Impacts of host phylogeny, diet, and geography on the gut microbiome of rodents
Source: PLoS One. 2025 Jan 16;20(1):e0316101. doi: 10.1371/journal.pone.0316101 (PMC11737772; doi:10.1371/journal.pone.0316101)
Supplement: S6 Table — (PDF) [file pone.0316101.s007.pdf]

S6 Table. Pairwise test results of the effect of host species, diet and locality on the microbiota composition based on unweighted UniFrac distances.

**Host species pairs**	**F.Model**	**R2**	**P-value**
C. hispidus vs G. attwateri	5.134004	0.46111	0.035*
C. hispidus vs N. floridana	3.369611	0.29637	0.006*
C. hispidus vs S. hispidus	4.105925	0.194539	0.001*
C. hispidus vs P. gossypinus	2.904833	0.26638	0.009*
C. hispidus vs G. breviceps	4.267796	0.415649	0.036*
C. hispidus vs N. leucodon	3.743991	0.348473	0.005*
C. hispidus vs P. nasutus	3.712127	0.346535	0.012*
C. hispidus vs P. truei	3.296003	0.3973	0.028*
C. hispidus vs N. mexicana	3.427657	0.328708	0.019*
C. hispidus vs P. boylii	2.969119	0.331038	0.034*
G. attwateri vs N. floridana	3.399919	0.298241	0.004*
G. attwateri vs S. hispidus	3.993105	0.19021	0.001*
G. attwateri vs P. gossypinus	2.846151	0.262411	0.008*
G. attwateri vs G. breviceps	3.024911	0.335173	0.039*
G. attwateri vs N. leucodon	3.20577	0.314113	0.008*
G. attwateri vs P. nasutus	3.243973	0.316671	0.008*
G. attwateri vs P. truei	3.356578	0.401669	0.025*
G. attwateri vs N. mexicana	3.131164	0.309063	0.011*
G. attwateri vs P. boylii	3.542708	0.371248	0.034*
G. breviceps vs N. leucodon	2.596914	0.270599	0.018*
G. breviceps vs P. nasutus	3.053111	0.303698	0.008*
G. breviceps vs P. truei	2.97642	0.373152	0.03*
G. breviceps vs N. mexicana	2.36829	0.252799	0.027*
G. breviceps vs P. boylii	3.255878	0.351763	0.026*
N. floridana vs S. hispidus	4.010915	0.174305	0.001*
N. floridana vs P. gossypinus	2.391143	0.192972	0.002*
N. floridana vs G. breviceps	3.096328	0.279041	0.006*
N. floridana vs N. leucodon	1.949074	0.178013	0.002*
N. floridana vs P. nasutus	2.692371	0.230267	0.003*
N. floridana vs P. truei	2.424367	0.257245	0.008*
N. floridana vs N. mexicana	2.433699	0.212853	0.002*
N. floridana vs P. boylii	2.690211	0.251652	0.007*
N. leucodon vs P. nasutus	2.625127	0.247068	0.007*
N. leucodon vs P. truei	2.470877	0.291691	0.016*
N. leucodon vs N. mexicana	1.779375	0.181952	0.015*
N. leucodon vs P. boylii	2.703921	0.278642	0.007*
N. mexicana vs P. boylii	2.819429	0.287128	0.023*
P. gossypinus vs G. breviceps	2.716832	0.253511	0.003*
P. gossypinus vs N. leucodon	2.454037	0.214251	0.003*
P. gossypinus vs P. nasutus	1.518402	0.144357	0.031*
P. gossypinus vs P. truei	1.439296	0.170547	0.044*

P. gossypinus vs N. mexicana	2.53114	0.219505	0.004*
P. gossypinus vs P. boylii	1.652378	0.171189	0.031*
P. leucopus vs C. hispidus	2.226069	0.156478	0.002*
P. leucopus vs G. attwateri	2.911479	0.195251	0.002*
P. leucopus vs N. floridana	2.469784	0.149958	0.001*
P. leucopus vs S. hispidus	3.645422	0.136812	0.001*
P. leucopus vs P. gossypinus	1.167854	0.076995	0.143
P. leucopus vs G. breviceps	2.695193	0.183406	0.002*
P. leucopus vs N. leucodon	2.452404	0.158707	0.001*
P. leucopus vs P. nasutus	1.748665	0.118564	0.011*
P. leucopus vs P. truei	1.191859	0.097759	0.139
P. leucopus vs N. mexicana	2.416947	0.156772	0.001*
P. leucopus vs P. boylii	1.501632	0.111219	0.043*
P. nasutus vs P. truei	1.303888	0.17852	0.188
P. nasutus vs N. mexicana	2.869501	0.263996	0.01*
P. nasutus vs P. boylii	1.647636	0.19053	0.083
P. truei vs N. mexicana	2.556173	0.298752	0.016*
P. truei vs P. boylii	0.900111	0.152558	0.714
S. hispidus vs P. gossypinus	3.467109	0.154319	0.001*
S. hispidus vs G. breviceps	3.80437	0.182864	0.001*
S. hispidus vs N. leucodon	3.578112	0.165821	0.001*
S. hispidus vs P. nasutus	3.723452	0.171402	0.001*
S. hispidus vs P. truei	2.942605	0.155343	0.002*
S. hispidus vs N. mexicana	3.680954	0.169778	0.001*
S. hispidus vs P. boylii	3.486138	0.170171	0.003*
**Dietary guild**			
Granivore vs Omnivore	2.637888	0.089004	0.002*
Herbivore vs Omnivore	3.928739	0.056997	0.001*
Herbivore vs Granivore	2.657339	0.056954	0.001*
**Locality**			
Blaine vs Cimarron	0.975441	0.039056	0.539
Cleveland vs Blaine	1.143357	0.222298	0.166667
Cleveland vs Cimarron	1.492158	0.050595	0.036*
Delta vs Love	1.581509	0.283348	0.066667
Delta vs Cleveland	1.152771	0.141396	0.249
Delta vs Blaine	0.889649	0.228722	1
Delta vs Cimarron	1.330008	0.046947	0.072
Fayette vs Hopkins	2.393438	0.06959	0.002*
Fayette vs Delta	1.200317	0.051737	0.142
Fayette vs Love	1.712732	0.078881	0.016*
Fayette vs Cleveland	1.322076	0.054357	0.087
Fayette vs Blaine	0.872357	0.043898	0.804
Fayette vs Cimarron	2.433776	0.053568	0.001*
Hopkins vs Delta	1.546501	0.088137	0.044*

Hopkins vs Love	1.954363	0.122497	0.006*
Hopkins vs Cleveland	1.624089	0.087204	0.029*
Hopkins vs Blaine	1.181262	0.083297	0.333
Hopkins vs Cimarron	3.018706	0.075432	0.001*
Love vs Cleveland	1.259518	0.201216	0.225
Love vs Blaine	2.601112	0.722308	0.333333
Love vs Cimarron	1.785184	0.066648	0.008*
